# ‘Investing’ in care for old age? An examination of long-term care expenditure dynamics and its spillovers

**DOI:** 10.1007/s00181-022-02246-0

**Published:** 2022-05-27

**Authors:** Joan Costa-Font, Cristina Vilaplana-Prieto

**Affiliations:** 1 London School of Economics and Political Science, CESIfo & IZA Bonn, Department of Health Policy, London School of Economics, Houghton Street, London, WC2A 2AE UK; 2 Department of Economic Analysis. Faculty of Economics and Business, University of Murcia, Murcia, Spain

**Keywords:** Long-term care spending, Panel-VAR, Dynamic panel data, Female labour market participation, Health spending, Care spillovers, I18, J1

## Abstract

We study the dynamic drivers of expenditure on long-term care (LTC) programmes, and more specifically, the effects of labour market participation of traditional unpaid caregivers (women aged 40 and older) on LTC spending, alongside the spillover effects of a rise in LTC expenditure on health care expenditures (HCE) and the economy (per capita GDP). Our estimates draw from a panel of more than a decade worth of expenditure data from a sample of OECD countries. We use a panel vector auto-regressive (panel-VAR) system that considers the dynamics between the dependent variables. We find that LTC expenditure increases with the rise of the labour market participation of the traditional unpaid caregiver (women over 40 years of age), and that such expenditures rise exerts large spillover effects on health spending and the economy. We find that a 1% increase in female labour participation gives rise to a 1.48% increase in LTC expenditure and a 0.88% reduction in HCE. The effect of LTC spending over HCE is mainly driven by a reduction in inpatient and medicine expenditures, exhibiting large country heterogeneity. Finally, we document significant spillover effects of LTC expenditures on per capita GDP.

**Supplementary Information:**

The online version contains supplementary material available at 10.1007/s00181-022-02246-0.

## Introduction

The efficient design of long-term care (LTC) programmes, providing support to older age disabled individuals in need of  assistance, is a forefront economic policy problem in many European countries (European Commission 2015). The constrained supply of traditional (unpaid or informal) care (Pezzin and Steinberg Schone 1999) and the expansion of the labour market participation of traditional unpaid caregivers are increasing the demand of formal LTC services. More specifically, it is possible to observe an increase in the demand for home care services, and to a lesser extent, nursing home care. However, how does the reduction in the supply of informal care, (measured by the increasing labour market participation of traditional caregivers, women over 40 years of age), impact the demand and, especially, long-term care expenditures? Does long-term care spending expansion reduce the use of public and private health care and boost economic activity?

The introduction of LTC programmes can impact health care as health care programmes are designed to fund only limited intensity of post-acute care following a hospital stay (Hermiz et al. 2002; Weaver and Weaver 2014; Sands et al. 2006), and hospital utilisation is sensitive to the inadequate subsidisation of LTC, which can be a source of inefficiencies such as bed blocking (Costa-Font et al. 2018; Rapp et al. 2015). Yet, to date, we still know little about the spillover effects of public LTC spending.

This paper is the first to examine the cross-country dynamic determinants of LTC expenditure (and its social and health components), to study the aggregate spillover effects on health care expenditure, as well as its different expenditure categories (such as outpatient, inpatient and medicine spending) and the economy. The availability of cross-national time-series data from OECD countries opens the possibility of studying the dynamic effects of the expansion of LTC expenditures. So far, the current estimates of LTC expenditure determinants are mostly descriptive (Colombo and Mercier 2012), and they tend to disregard the important persistence in expenditure, as well as causality claims (Costa-Font et al. 2016; De la Maisonneuve and Martins 2013).

This paper exploit the effect of changes in the labour participation of traditional unpaid caregivers (women over 40 years of age) on LTC spending and Gross Domestic Product (GDP) per capita, alongside the subsequent spillover effects on health expenditure and the economy (GDP per capita). Our estimates are drawn from a panel-VAR (panel-VAR) GMM model on a sample of OECD countries for the period 2002 to 2015. Unlike previous studies, we consider several dynamic pathways, the endogenous interaction between female labour participation (of women over 40 years of age), LTC expenditure, as well as the effect of LTC expenditure on both HC expenditure, and GDP per capita. The main advantage of the panel-VAR model is that all variables are treated as endogenous and consider unobserved individual heterogeneity including country-specific fixed effects, which improves the consistency of the model (Love and Zicchino 2006).^1^ Finally, we complete our analysis with the estimation of Bayesian panel-VAR with partial pooling for the groups of Northern and Southern countries. This approach allows us to deal with the reduced panel dimension as well as with interdependencies among countries.^2^

Our main results reveal the following. First, we find a significant effect of labour market participation of traditional unpaid caregivers (women over 40 years of age) on LTC expenditures. We estimate that a 1% increase in female labour participation leads to an increase of LTC expenditure by 1.48% in the subsequent period. Second, we find evidence of caregiving spillovers’ on health spending which are driven by a reduction in inpatient and medicine expenditure. Third, we document the effect of LTC expenditure on health spending and per capita GDP. We document that a 1% increase in LTC expenditures in one period increases per capita GDP by 0.20% in the next period but reduces HCE by 0.6% in such a period (mainly due to a reduction in medicine expenditure by 0.86% and inpatient expenditure by 0.50%).

Next, we relate our paper to the existing literature. Section three describes the data and empirical strategy. Section four reports the results, and a final section concludes with a discussion and policy implications.

## Related literature

### Health and long-term care expenditure estimates

LTC services share some characteristics with heavily subsidised health care, yet unlike health care, personal LTC can be delivered by unpaid household members informally.^3^ However, evidence suggests large heterogeneity across the OECD countries in both caregiving duties and formal care provision of services. Descriptive analysis suggests that long-term care spending is associated with population ageing, female labour market participation, and the institutionalization of the care service system (Olivares-Tirado et al. 2011; Costa-Font et al. 2015; Huei-Ru et al. 2016).^4^ Costa-Font et al. (2015) estimate an income elasticity of 3.2, indicating a high sensitivity of per capita public LTC expenditure to a change in a country’s per capita GDP.^5^

### Female labour market participation

Given that informal care is still the most common form of care for old age in almost all countries, if formal and informal care are substitutes (Picone and Wilson 1999; Stern 1995; Carmichael and Charles 2003; Carmichael et al. 2010; Nizalova 2012), a contraction in the supply of informal care (resulting from the expansion of labour market participation of traditional caregivers) can rise the demand for paid care.^6^

### Health system spillovers

A higher LTC utilisation can exert spillover effects on the health system and, especially on costlier hospital care utilisation (Hofmarcher et al. 2007; Bodenheimer 2008; Mur-Veeman and Govers 2011). Some evidence already documents that the introduction of home care programs reduced delays in hospital discharges and emergency readmissions (Hermiz et al. 2002; Sands et al. 2006; Weaver and Weaver 2014).^7^ Hence, the expansion of LTC services can give rise to a reduction in health care utilisation.

### Spillovers on to the economy

The expansion of public LTC spending can give rise to a subsequent effect on economic activity by boosting the ‘care sector’. Previous studies such as De Henau et al. (2016) show that the care economy may raise GDP growth more than investment in construction.^8^ The next section describes the empirical strategy used to estimate the determinants of LTC expenditure and its determinants.

## Empirical strategy and data

### Empirical strategy

The identification of the drivers of LTC expenditure, such as labour market participation of traditional unpaid caregivers (women over 40 years of age), and its spillover effects on health care expenditures faces several methodological challenges, including potential reverse causality and omitted variables bias, as well as both time and cross-sectional correlation, and such dynamic effects need to be modelled. Relative to conventional time series modelling, the panel-VAR model considers the heterogeneity of the cross-sectional dynamics, which provides more information about the sources of heterogeneity in the system. The panel-VAR specification exploits the temporal and cross-section dimension of the data to infer dynamic relations between the dependent variables,^9^ allowing all covariates to be treated as fully endogenous while simultaneously modelling the unobservable heterogeneity through fixed effects (which account for time-invariant characteristics intrinsic to each country). Hence, resulting in an improvement in the consistency of the estimation (Love and Zicchino 2006).

So far, no previous study has used a sample of panel data over a considerable period for a large number fo countries, accounting for the fact that the presence of cross-sectional dependence in panel data may compromise the stationarity of the variables under analysis.^10^ To our knowledge, no previous study has examined the dynamic impact of healthcare expenditure and GDP of a LTC expenditure shocks.^11^

In a panel-VAR model specification, each variable is explained by its own lag, the lagged values of the other system variables and individual country-specific terms. Treating all variables as endogenous prevents us from using weak instruments. An additional advantage of a panel-VAR model is that it allows goodness of fit analysis and observing the reaction to different shocks. The specification of a panel-VAR model of p order proposed by Canova and Cicarrelli (2004) is as follows^1^:

1 $$Y_{i,t} = {\rm A}\left( l \right)_{i0} + {\rm A}\left( l \right)_{i1} Y_{i,t - 1} + \varepsilon_{i,t}$$

where all variables of vector $Y_{t}$ are considered endogenous, allowing for a joint dynamic analysis. If we denote for *q* the number of endogenous variables, then vector $Y_{t}$ has dimension *q* × 1. In turn, $Y_{t}$ contains a cross section dimension, $y_{i,t} = \left( {y_{1,t}^{\prime } ,y_{2,t}^{\prime } , \ldots ,y_{N,t}^{\prime } } \right)$, where *i* = 1,2,….,*N* indicates the number of countries and *t* = 1,2,…,*T* indicates the number of years observed for each country. The fixed effects (${\rm A}\left( l \right)_{i0}$) are captured by a *q* × 1 vector, where *l* is a polynomial in the lag operator such that ${\rm A}\left( l \right)_{i0} = \mathop \sum \nolimits_{i = 0}^{N} A_{i} l^{j} , j = 1,2, \ldots ,p$.

The term ${\rm A}\left( l \right)_{i1}$ is a *q* × *N* matrix of lagged coefficients and $\varepsilon_{i,t} = (\varepsilon_{1,t} , \varepsilon_{2,t} , \ldots ,\varepsilon_{N,t} )\prime$ is the error term with a zero-mean, with variance–covariance matrix independent of t and such that $\varepsilon_{i,t}$ of different periods are independent of each other: $\varepsilon_{i,t} \sim iid\left( {0,\Sigma } \right)$. In this paper, we estimate the following two panel-VAR models to measure the effect of labour market participation on long-term care expenditure, and a second specification for the effect of long-term care expenditure spillovers on health care spending.

Estimating LTC expenditures and female labour market effects, we consider a first specification containing three equations for which the dependent variables are $Y_{i,t} = \left\{ {{\text{FemPart}}_{i,t} , {\text{LTC}}_{i,t} , {\text{GDPpc}}_{i,t} } \right\}$. ${\text{FemPart}}_{i,t}$ measures the labour market participation rate of the traditional caregiver (women 40 years and older), ${\text{LTC}}_{i,t}$ refers to public long-term care (LTC) expenditures in per capita terms, and ${\text{ GDPpc}}_{i,t}$ measures gross domestic product per capita. Besides, LTC expenditures can exert heterogeneous effects on different health care spending categories. We distinguish between the three types of LTC expenditures available (total, health-related services, and social-related services).^12^

The underlying assumption of our dynamic model is that the labour participation of the traditional caregiver (women over 40 years of age) increases LTC expenditures (via higher use of formal care), which in turn expands GDP pc. However, such effects might differ across countries. In our data, we can, consistently with Reher (1998) and Kohli et al. (2005), differentiate between Southern European countries (with strong family ties) from Northern European countries (with weaker family ties).The direct relationship between LTC expenditures and GDP pc results from the combination of community-based services, residential care and support to informal caregivers (e.g. respite services), which in turn, can constitute an important source of employment, and consequently, of economic growth in the years to come (Spasova et al. 2018). Ikkaracan and Kim (2019) performed a macroeconomic simulation study with data from 45 countries to compute the amount of employment needed to meet specific Sustainable Development Goals by 2030. They found that the long-term care sector would require the creation of 29.6 million jobs. Specific country projections suggest that the demand for formal caregivers (home care and residential homes) would increase by 980,000 new workers in 2050 for Australia (Mavromaras et al. 2017) and would lie between 16 and 26 thousand employees in 2030 for Poland (Golinowska et al. 2014).

Next, we formulate a second specification that considers three equations in which the dependent variables are $Y_{i,t} = \left\{ {{\text{LTC}}_{i,t} ,{\text{HC}}_{i,t} , {\text{GDPpc}}_{i,t} } \right\}$, and more specifically public LTC expenditures in per capita terms ${\text{LTC}}_{i,t}$, public healthcare expenditures in per capita terms ${\text{HC}}_{i,t}$, and gross domestic product per capita ${\text{ GDPpc}}_{i,t}$. The previous empirical literature has shown that LTC spending may help reduce the onset of unmet needs and, hence, reduce health utilisation (Allen and Moor 1997; Desai et al. 2001; Lima and Allen 2001). Similarly, an early post-discharge period after hospitalisation results in approximately 20% of complications that involve re-hospitalisation (Foster et al. 2003; Naylor et al. 2007), although these adverse effects can be avoided with more formal support (home care). LTC spending can in turn facilitate a smoother transition from hospital to home. Likewise, health care expenditures (HCE) can impact on per capita GDP given that some effective interventions improve health, which in turn can exert subsequent effects on labour supply and productivity, boosting GDP.^13^

Our panel data specification imposes as a restriction that the coefficients ${\rm A}\left( l \right)_{i1}$ are equal for all countries, though we add country fixed effects (${\rm A}\left( l \right)_{i0}$) to our specification to control for time-variant cross-country effects. Nevertheless, one of the limitations of including the fixed effects is that they are usually correlated with the regressors throughout the lag of the dependent variables (Blundell and Bond 1998). The Helmert transformation, which consists of applying forward mean differencing,^14^ is used to maintain the orthogonality between the regressors and their lags, allowing the mentioned lags to be used as instruments. Furthermore, GMM method is used for efficiency purposes (Holtz-Eakin et al. 1988). To determine the lag structure, we draw on Hansen’s J statistic^15^ and Andrews and Lu (2001) who proposed the use of consistent Moment and Moment Selection Criteria (MMSC). Additionally, we verify the stability condition of the model (Hamilton 1994)^16^ we perform a battery of Granger causality tests to determine whether variable $Y_{1t}$ carries any information about another variable ($Y_{2t}$). The results of these tests help us to establish a causal order of the variables in the system.^17^

Finally, we distinguish between Northern and Southern countries, considering the interdependencies among countries in each group. The latter reduces considerably the dimension of the panel. To overcome this limitation, we rely on the estimation of Bayesian panel-VAR model (Doan et al. 1984; Canova and Ciccarelli 2004; Koop and Korobilis 2019). In the Bayesian panel-VAR model, the parameters are assumed to be random variables, characterized by an underlying probability distribution (Doan et al. 1984). To consider the full interdependencies between Northern and Southern European countries (in their respective panels), we draw upon a partial pooling analysis (Canova and Ciccarelli 2013).

### Data

We use a panel dataset that covers 27 countries for the period 2002–2015.^18^ We exploit time and cross-country variation, whereas the estimations for the group of Northern and Southern countries have been computed for the sub-period 2009–2015, due to data limitations.^19^ The country sample has been selected to maximize the number of years with available information (see Table A2). All variables used in the econometric analysis come from the OECD Health Data and Social Protection Data for OECD countries and are listed in Table A3. All economic variables are measured in US dollars, constant prices, are adjusted for purchasing power parities (PPP) and refer only to public expenditure (e.g. government or compulsory schemes). We use GMM panel-VAR with one lag and one to four instruments for estimations using the whole sample, but due to the reduced time dimension of the panel for Northern and Southern countries, we have employed Bayesian panel-VAR estimation.

Dependent variables (see Table A1).

- LTC expenditure per capita includes a range of medical and personal services that are consumed with the primary goal of alleviating pain and reducing or managing deterioration in health status among individuals with a degree of long-term dependency OECD 2018).^20^ We distinguish between total, medical^21^ and social LTC spending.^22^
- Health care expenditure per capita (OECD 2017). We differentiate between total health care expenditure excluding LTC (related to health services),^23^ inpatient and outpatient care, and pharmaceuticals.
- GDP per capita (seasonally adjusted), female labour participation (40 years and older).

The coverage and comparability of LTC spending estimates have improved with the implementation of a “System of Health Accounts 2011” (OECD 2017) which provides a framework for the measurement of health and LTC spending. However, in-depth analyses of data submissions suggested that full comparability of LTC spending data across OECD countries is still hampered to some extent (Mueller and Morgan 2017).^24^

Figure A1 (Appendix A) depicts a linear association between per capita GDP and LTC spending, with a flattening out effect explained by two country outliers, namely Luxembourg and Norway. This evidence is consistent with the hypothesis that LTC is a normal good, and its investment increases with a country’s economic development. Figure A2 shows that consistent with expectations, female labour market participation exerts a steep effect on LTC spending. Finally, in Figure A3, we find evidence of a positive association between health spending and LTC spending, though it tails up at higher levels of spending.

Pre-estimation tests. To estimate a panel-VAR model, an important condition is for the variables to be stationary.^25^ Accordingly, we employ two-unit root tests for panel data to test for stationarity, namely the Harris-Tzavalis (1999) and Im-Pesaran-Shin (2003) tests. The null hypothesis is that panels contain unit roots and are stationary. Importantly, the results of both tests (see Table B1) strongly suggest that all the variables (LTC expenditures, HCE, female labour participation and GDP pc expressed in logarithms) do not follow a unit root process. Hence, non-stationarity is not a concern in our estimates. However, one potential concern is the presence of possible cross section autocorrelation resulting from common factors (Levin et al. 2002). In those cases, we subtract the average of the group at each time for each time series.

## Results

### Determination of panel-VAR length

The decision on the order length of the panel-VAR is based on the tests of MMSC proposed by Andrews and Lu (2001).^26^ Table B2 illustrates the results of the MMSC and Hansen’s J statistic using four instruments for each of the endogenous variables (from the first to the fourth lagged dependent for both models). The results are shown using one, two, and three lags, respectively. In all cases, the model with one lag is the one that simultaneously minimises the three criteria and corroborates the suitability of the instruments used.

### Validation of the panel-VAR model

After performing the Granger causality Wald tests for each equation of the underlying panel-VAR model, we examine the stability condition of panel-VAR. The results of the Granger causality test are displayed in Table B3, although predicted associations should be considered with reservation and tested with additional analysis.

### Variance decomposition of forecast error

Tables B5 and B6 show the variance decomposition of the forecast error for the two proposed models (including different variants depending on the use of the different types of LTC expenditures and HCE) and different groups of countries.

Variance error decomposition for long-term care expenditures suggest that female labour participation (of women over 40 years of age) explains about 80% of Forecast Error Variance (FEV) of LTC expenditures in Nordic countries for both total and health- and social-related expenditures. This percentage is much higher than the one observed for all countries (1.9% for total LTC, 6.5% for health LTC, and 16.7% for social LTC) and especially for Southern countries (3.8%, 15.7%, and 14.9%, respectively). In contrast, social LTC expenditures explain 12.1% of the FEV of female labour participation in Nordic countries, compared with a percentage lower than 1% in the group of all countries and Southern countries. Finally, when we examine total LTC expenditures, we find that it explains 23.9% of the FEV of total health expenditures for the group of all countries—an amount below the 70.3% of Nordic countries. For the entire sample, LTC expenditures explain 19.71% of the FEV, in contrast to 3.91% for social LTC expenditures.^27^

Variance error decomposition for caregiving spillovers suggest that total HCE explains 5.8% of the FEV of total LTC expenditures, and 3.47% in Southern countries, but it is not significant for all countries. On the other hand, an LTC shock accounts for 5.11% of the variation of HCE for all countries (19.71% in Nordic countries, 4.48% in Southern countries). Similarly, LTC shocks explain a higher percentage of the variation in outpatient, inpatient and medicine expenditure in Nordic countries. A result of particular interest is the higher contribution of LTC shock to the variance of GDP pc: 18.88% for all countries, 27.63% for Nordic countries and 15.35% for Southern countries (as compared to the effect of HCE shock: not significant for all countries, 2.61% for Nordic countries and 3.32% for Southern countries).

### Model estimates

#### Female labour participation effects on LTC expenditure and per capita GDP (GDP pc)

Table 1 reports the results of the panel-VAR model for the female participation rate, LTC expenditures (total, health, and social) and GDP pc. We display the estimates for all countries in the first column, Northern countries in the second one and Southern countries in the last one. We find that a 1% increase in the female participation rate in one period raises total LTC expenditures by 1.48% during the following period. However, in Northern countries, the response of LTC expenditures is almost four times as large (3.96%), whilst it is not significant in Southern countries.

**Table 1:** Panel-VAR for female labour participation, LTC expenditure and GDP pc

	LTC Expenditure (in logs)			Health LTC Expenditure (in logs)			Social LTC Expenditure (in logs)		
	All countries	Northern European Countries	Southern European countries	All countries	Northern European Countries	Southern European countries	All countries	Northern European Countries	Southern European countries
*Eq: LogFempart*									
LogFempart(− 1)	0.651***	1.677***	0.812	0.642***	1.363***	0.825***	0.684***	1.207***	0.847*
	(0.063)	(0.121)	(1.362)	(0.058)	(0.194)	(0.087)	(0.062)	(0.122)	(0.500)
LogLTC(− 1)	0.007	0.051***	1.373	0.008***	0.039	0.047*	− 0.004	0.008***	0.056
	(0.005)	(0.015)	(7.338)	(0.003)	(0.028)	(0.027)	(0.003)	(0.003)	(0.034)
LogGDP pc(− 1)	0.127***	0.047**	0.294	0.129***	0.077**	0.098	0.127***	0.016	0.079
	(0.024)	(0.022)	(1.545)	(0.023)	(0.031)	(0.185)	(0.025)	(0.024)	1.456)
*Eq: LogLTC*									
LogFempart(− 1)	1.479**	3.957***	3.426	1.514***	3.810***	0.818	1.420***	4.888***	3.575**
	(0.438)	(0.874)	(3.162)	(0.434)	(1.730)	(0.603)	(0.527)	(0.812)	(1.589)
LogLTC(− 1)	0.188***	− 0.510***	1.079***	− 0.036	− 0.179	0.560***	0.298***	− 0.244***	0.920***
	(0.057)	(0.115)	(0.364)	(0.064)	(0.699)	(0.099)	(0.089)	(0.042)	(0.292)
LogGDP pc(− 1)	0.810***	3.213***	7.462*	0.357*	1.863**	2.415**	1.009***	7.472***	4.281**
	(0.226)	(0.373)	(3.966)	(0.210)	(0.786)	(0.996)	− (0.261)	(0.307)	(1.873)
*Eq: LogGDP pc*									
LogFempart(− 1)	0.186*	0.643***	0.053	0.141	0.688***	0.674	0.320***	0.329***	0.213
	(0.110)	(0.228)	(0.141)	(0.095)	(0.190)	(0.429)	(0.114)	(0.119)	(0.132)
LogLTC(− 1)	0.238***	1.053***	0.590**	0.172***	0.859***	0.0820***	0.055**	0.114***	0.488***
	(0.013)	(0.014)	(0.035)	(0.038)	(0.0209)	(0.004)	(0.021)	(0.024)	(0.120)
LogGDP pc(− 1)	0.514***	0.069**	0.241	0.550***	0.050	1.308	0.516***	0.322***	0.153
	(0.061)	(0.028)	(0.304)	(0.074)	(0.046)	(1.225)	(0.077)	(0.028)	(0.158)
*N*	350	28	28	350	28	28	350	28	21
Criterion function	0.234	0.606	0.398	0.238	0.604	0.498	0.230	0.702	0.454
Hansen’s J statistic	53.616	21.818	8.763	52.923	21.758	12.443	52.647	25.267	9.988

Note: Dependent variables in logarithms. Helmert transformation applied before estimation. Heteroscedasticity and serial correlation robust standard errors between parenthesis. ***, ** and ** denote significance at the 1%, 5% and 10% significance level, respectively

Estimations for all countries: 2002–2015. Estimation of GMM panel-VAR with one lag and one to four lags in the endogenous instruments. Estimations for Northern countries and Southern countries: 2009–2015. Northern countries include Denmark, Finland, Norway and Sweden. Southern countries include Greece, Italy, Portugal and Spain. LTC_Social not available for Greece. Estimation of Bayesian panel-VAR. Standard errors are obtained after 100 Monte Carlo repetitions

Next, we distinguish between health and other components, and we document that a 1% increase in female labour market participation increases the health component of LTC spending by 1.5% and the social component by 1.42% for the group of all countries. Finally, and consistent with the evidence of ‘care economy effects’, we document that a 1% increase in LTC total expenditures gives rise to a 0.2% GDP pc increase in the next period for the entire sample, yet this effect is larger among in Northern countries (1.05%) than in Southern countries (0.59%). Such results are explained by the presence of supply constraints in several countries as caregivers’ wages fall at the lower end of the pay distribution. Yet, a rise in caregivers’ wages gives rise to a subsequent GDP rise. Although the magnitude of these figures may seem large, the results are consistent when compared with those of De Henau et al. (2016), who examine the multiplier effect of increased spending in the care sector on the economy. If 2% of GDP was invested in the care industry, total GDP would grow between 7.7% (US) and 4.8% (Denmark).

The health component of LTC drives the overall effect, and that of Northern countries, but in Southern countries, it is channelled mainly through the LTC social component. Finally, we find that an increase of 1% in LTC expenditures in one period raises the female participation rate by 0.051% in the subsequent period in Northern countries, although it is not significant for the total of the sample or for Southern countries.

#### LTC expenditure effects on health and per capita GDP

Table 2 provides the results of the panel-VAR model for total LTC expenditures, HC expenditures, and per capita GDP. We find that a 1% increase in LTC expenditure during one period gives rise to a 0.6% reduction in HCE in the subsequent period. The reduction for the group of Nordic European countries is almost three times the average (1.78%). In contrast, the effect is estimated to be slightly smaller, 0.55% among the group of Southern European countries.

**Table 2:** Panel-VAR for total HC expenditure

	LTC Expenditure (in logs)			Health LTC Expenditure (in logs)			Social LTC Expenditure (in logs)		
	All countries	Northern European Countries	Southern European countries	All countries	Northern European Countries	Southern European countries	All countries	Northern European Countries	Southern European countries
*Eq: LogLTC*									
LogLTC(− 1)	0.8648***	1.5684***	2.3241*	0.9691***	0.9448***	0.8064***	0.5635***	2.1541***	0.8769***
	(0.0698)	(0.1083)	(1.603)	(0.0756)	(0.0723)	(0.0496)	(0.1460)	(0.7493)	(0.1226)
LogHC total(− 1)	− 0.0336	− 0.3076***	− 0.6569***	− 0.2005	− 0.0464	− 0.5699**	− 0.0378	− 2.2273***	− 0.7037**
	(0.0240)	(0.0303)	(0.2162)	(0.1793)	(0.1149)	(0.2785)	(0.0235)	(0.398)	(0.3242)
LogGDP pc(− 1)	0.0327	0.4499***	0.2750*	0.0429	0.1949	0.4068	0.0318	3.4885***	2.1150**
	(0.0381)	(0.0516)	(0.1537)	(0.3134)	(0.1577)	(0.5796)	(0.0343)	(0.6606)	(1.2213)
*Eq: LogHC total*									
LogLTC(− 1)	− 0.6033***	− 1.7852***	− 0.5558**	− 0.2352***	− 1.6909***	− 0.1530***	− 0.3941***	− 0.1017***	− 0.3988***
	(0.1851)	(0.6076)	(0.2404)	(0.0636)	(0.5055)	(0.0189)	(0.0969)	(0.0238)	(0.1350)
LogHC total(− 1)	0.5377***	2.2399***	0.4728***	0.8229***	1.2554***	0.7442***	0.8791***	0.7584***	− 0.9736*
	(0.0765)	(0.1950)	(0.1212)	(0.0888)	(0.0166)	(0.1099)	(0.0991)	(0.1275)	(0.5604)
LogGDP pc(− 1)	0.4731***	2.1770***	0.0424	0.0775	0.8929***	0.1844	0.0042	0.5827***	1.0492*
	(0.0780)	(0.2013)	(0.2589)	(0.1844)	(0.1378)	(0.2233)	(0.2054)	(0.0494)	(0.5819)
*Eq: LogGDP pc*									
LogLTC(− 1)	0.2034***	1.0891**	0.6111***	0.1856***	0.8480***	0.0831***	0.0536***	0.1312***	0.5018***
	(0.0569)	(0.4504)	(0.1258)	(0.0534)	(0.0497)	(0.0222)	(0.0149)	(0.0346)	(0.0680)
LogHC total(− 1)	0.0294	0.8222***	0.3006***	0.0062	0.3032***	0.4611*	0.0544	0.2781	− .2052
	(0.0703)	(0.1447)	(0.0570)	(0.0569)	(0.0581)	(0.2595)	(0.0386)	(0.1932)	(0.2421)
LogGDP pc(− 1)	0.5331***	0.0711**	0.3427	0.5650***	0.0707	1.3234	0.5472***	0.3331***	0.2019
	(0.0632)	(0.0314)	(0.3047)	(0.0651)	(0.0558)	(2.3201)	(0.0812)	(0.0367)	(0.2150)
*N*	350	28	28	350	28	28	336	28	21
Criterion function	0.2267	0.7993	0.7628	0.2312	0.7792	0.8197	0.2191	0.7342	0.4731
Hansen’s J statistic	417.074	287.736	152.552	529.471	280.497	163.947	466.582	293.697	99.353

Note: Dependent variables in logarithms. Helmert transformation applied before estimation. Heteroscedasticity and serial correlation robust standard errors between parentheses. ***, ** and ** denote significance at the 1%, 5% and 10% significance level, respectively

Estimations for all countries: 2002–2015. Estimation of GMM panel-VAR with one lag and one to four lags in the endogenous instruments. Estimations for Northern countries and Southern countries: 2009–2015. Northern countries include Denmark, Finland, Norway and Sweden. Southern countries include Greece, Italy, Portugal and Spain. LTC_Social not available for Greece. Estimation of Bayesian panel-VAR. Standard errors are obtained after 100 Monte Carlo repetitions

When we disentangle the effect by types of care, we find that social LTC expenditures are the main driver of HCE reductions in the entire sample and for Southern countries. In contrast, we do not find large reverse effects: a 1% increase in HCE in one period reduces total LTC expenditures in the following period by a small amount (− 0.31%) in Northern countries, and comparatively, this reduction is six times lower than the effect of total LTC expenditures on HCE. The opposite occurs in Southern countries, for whom an increase of 1% in HCE reduces total LTC expenditures in the subsequent period by 0.66%, which is more than the opposite effect.

Finally, and consistently with the productive effects of a care economy, we document that a 1% increase in total LTC expenditure increases GDP pc by 0.20% in the next period. This increase is mainly driven by health component of LTC expenditure in all countries and Northern countries, and by social LTC expenditure in Southern countries. In contrast, a 1% increase in HCE does not affect GDP pc in the next period for the total sample.^28^

- Inpatient expenditure
  Table 3 reports the results for the panel-VAR model that includes LTC expenditures, inpatient health care expenditures, and per capita GDP. We document that a 1% increase in LTC expenditures reduces inpatient expenditures by 0.5% for all countries, which is driven by social LTC expenditures.
  In contrast, an increase of 1% in inpatient expenditures leads to a smaller reduction in total LTC expenditures by 0.31% for the entire sample. As for GDP pc, we find that a 1% increase in total LTC expenditure increases GDP pc in the next period by roughly 0.2% for all countries, but an increase in inpatient expenditure does not affect per capita GDP. Yet, the effect in Northern (Southern) countries is 1.5 (1.41) times higher compared to an equivalent increase in inpatient expenditure.
- Outpatient expenditure
  The results of the panel-VAR model for outpatient expenditures are reported in Table 4. They suggest that a 1% increase in LTC expenditures only delivers small and significant effects in Northern European countries (a reduction in outpatient expenditures by 0.26% in the subsequent period). When we distinguish the type of LTC spending, we find that a 1% increase in health LTC expenditures leads to a reduction in outpatient expenditures by 0.12% in Northern countries and 0.12% in Southern countries. Similarly, we find that a 1% increase in outpatient expenditures reduces health LTC expenditures in Southern countries (− 2.73%), and in social LTC expenditures in Northern countries (− 1.93%). As expected, we find that both LTC and outpatient expenditure exert a positive effect on GDP pc, but the effect of the former is 2.65 times higher than the latter. Health related LTC expenditure is the main responsible driver of the boost in GDP pc in Northern countries, whereas social LTC expenditure is the main driver in Southern countries.
- Medicine expenditure
  Table 5 reports the results of the panel-VAR model for LTC expenditures, medicines expenditures and GDP pc. We find that a 1% increase in LTC reduces medicine spending by 0.87% in the whole sample and by 1.12% in Northern countries. This reduction is driven mainly by social LTC expenditures (0.8956% and 1.0675%, respectively). In contrast, a 1% increase in medicine expenditures exerts negligible effects on LTC expenditures (− 0.01% for all countries and − 0.04% for Northern countries). As for economic growth, the effect of a 1% increase in LTC expenditure over GDP pc is almost 5 times (0.2778% vs. 0.0580%) higher than an equivalent increase in medicine expenditure. Such a difference rises eight-fold when evaluated only among the sample of Northern countries.

**Table 3:** Panel-VAR for inpatient expenditure

	LTC Expenditure (in logs)			Health LTC Expenditure (in logs)			Social LTC Expenditure (in logs)		
	All countries	Northern European Countries	Southern European countries	All countries	Northern European Countries	Southern European countries	All countries	Northern European Countries	Southern European countries
*Eq: LogLTC*									
LogLTC(− 1)	0.9181***	1.7957***	0.7167***	0.9538***	1.0612***	0.6269***	0.9277***	10.5031***	–
	(0.0835)	(0.1107)	(0.1571)	(0.0661)	(0.0885)	(0.0400)	(0.2166)	(3.9341)	–
LogInpatient(− 1)	− 0.3091***	− 0.2042***	− 1.1032**	− 0.3043**	− 0.1860	− 1.1450***	− 0.3187***	− 1.5989***	–
	(0.0183)	(0.0300)	(0.0456)	(0.1256)	(0.1509)	(0.0980)	(0.0525)	(0.2403)	–
LogGDP pc(− 1)	0.0089	0.2693***	0.0647	0.1664	0.1362	0.1801	0.1161	7.4449***	–
	(0.0289)	(0.0713)	(0.1505)	(0.3092)	(0.2302)	(0.8546)	(0.0712)	(1.1103)	–
*Eq: LogInpatient*									
LogLTC(− 1)	− 0.5054**	− 1.3693***	− 1.0668***	− 0.0261*	− 0.4231***	− 0.8717***	− 0.5076***	− 0.9670***	–
	(0.2303)	(0.3382)	(0.1404)	(0.0143)	(0.0050)	(0.0129)	(0.1766)	(0.1416)	–
LogInpatient(− 1)	0.6831***	1.5811***	0.6020***	0.6456***	1.0938***	0.6990***	0.5043***	1.3235***	–
	(0.0591)	(0.0701)	(0.0399)	(0.0602)	(0.0350)	(0.0117)	(0.0774)	(0.1224)	–
LogGDP pc(− 1)	0.5016***	1.0282***	0.3514	0.5144***	1.1583***	0.4422*	0.5125***	0.7726***	–
	(0.1245)	(0.2477)	(0.2806)	(0.1229)	(0.0802)	(0.2383)	(0.1101)	(0.1150)	–
*Eq: LogGDP p*									
LogLTC(− 1)	0.1977***	1.0557***	0.4733***	0.1788***	0.7359***	0.0681***	0.0602***	0.1627***	–
	(0.0545)	(0.3466)	(0.0591)	(0.0488)	(0.0365)	(0.0188)	(0.0128)	(0.0434)	–
LogInpatient(− 1)	0.0277	0.6972***	0.3352**	0.0117	0.3255***	0.1101***	0.1321*	0.3171***	–
	(0.0471)	(0.0954)	(0.1155)	(0.0541)	(0.0697)	(0.0207)	(0.0790)	(0.1060)	–
LogGDP pc(− 1)	0.5611***	0.0972***	0.4506	0.5791***	0.1306	1.2289	0.5942***	0.4138***	–
	(0.0844)	(0.0156)	(0.3107)	(0.0868)	(0.1010)	(1.1029)	(0.1121)	(0.0754)	–
*N*	322	28	21	322	28	21	308	28	–
Criterion function	0.2028	0.8045	0.6078	0.1888	0.5502	0.5043	0.2138	0.7117	–
Hansen’s J statistic	425.874	289.603	121.559	396.500	198.074	100.866	416.874	284.697	–

Note: Dependent variables in logarithms. Helmert transformation applied before estimation. Heteroscedasticity and serial correlation robust standard errors between parentheses. ***, ** and ** denote significance at the 1%, 5% and 10% significance level, respectively

Estimations for all countries: 2002–2015. Estimation of GMM panel-VAR with one lag and one to four lags in the endogenous instruments. Estimations for Northern countries and Southern countries: 2009–2015. Northern countries include Denmark, Finland, Norway and Sweden. Southern countries include Greece, Italy, Portugal and Spain. LTC_Social not available for Greece and inpatient expenditure not available for Italy. Estimation of Bayesian panel-VAR. Standard errors are obtained after 100 Monte Carlo repetitions

**Table 4:** Panel-VAR for outpatient expenditure

	LTC Expenditure (in logs)			Health LTC Expenditure (in logs)			Social LTC Expenditure (in logs)		
	All countries	Northern European Countries	Southern European countries	All countries	Northern European Countries	Southern European countries	All countries	Northern European Countries	Southern European countries
*Eq: LogLTC*									
LogLTC(− 1)	0.9287***	1.5835***	0.9763***	0.9908***	0.9873***	1.1446***	0.6475***	1.4169***	0.8272***
	(0.0697)	(0.1643)	(0.2110)	(0.0646)	(0.0950)	(0.0479)	(0.1088)	(19.154)	(0.0680)
LogOutpatient(− 1)	− 0.0006	− 0.0341***	− 0.5278***	− 0.1851	− 0.0477	− 2.7341***	0.3882***	− 1.9360***	− 0.2091
	(0.0106)	(0.0122)	(0.0469)	(0.1207)	(0.0513)	(0.4085)	(0.0938)	(0.6351)	(0.1365)
LogGDP pc(− 1)	0.0287	0.1236**	0.0878	0.0509	1.5792***	0.9805	0.7016*	3.6841***	1.6601
	(0.0260)	(0.0599)	(0.2100)	(0.2847)	(0.1838)	(0.6496)	(0.3587)	(1.527)	(0.9803)
*Eq: LogOutpatient*									
LogLTC(− 1)	− 0.0136	− 0.2646***	− 0.1269	− 0.0250	− 0.1262***	− 0.1190***	− 0.1190*	− 0.1730***	− 0.0900*
	(0.4525)	(0.0158)	(0.4910)	(0.0285)	(0.0050)	(0.0398)	(0.0654)	(0.0529)	(0.0535)
LogOutpatient(− 1)	0.8672***	0.2583***	0.3742**	0.9557***	1.1138***	0.1112	1.1761***	1.4395***	− 0.0177
	(0.1245)	(0.0459)	(0.1522)	(0.1025)	(0.0331)	(0.1377)	(0.0641)	(0.1621)	(0.0966)
LogGDP pc(− 1)	0.0326	0.2443	0.0547	0.1045	0.5145***	− 0.1949	0.2323	− 0.0679	2.6602***
	(0.2717)	(0.1694)	(0.2404)	(0.2864)	(0.1948)	(0.3569)	(0.1792)	(0.0899)	(0.4308)
*Eq: LogGDP pc*									
LogLTC(− 1)	0.2101***	0.9827***	0.6286***	0.2215***	0.8257***	0.0866***	0.0635***	0.1428***	0.5190***
	(0.0540)	(0.2559)	(0.1506)	(0.0483)	(0.0589)	(0.0294)	(0.0146)	(0.0349)	(0.0538)
LogOutpatient(− 1)	0.0790**	0.1074***	0.0149	0.0527	0.0740**	0.5726**	0.0858***	0.1709***	0.0390
	(0.0359)	(0.0275)	(0.0434)	(0.0324)	(0.0306)	(0.2493)	(0.0272)	(0.0333)	(0.0255)
LogGDP pc(− 1)	0.5234***	0.0838**	0.3481	0.5154***	0.0826	1.3655	0.5829***	0.3494***	0.2016
	(0.0926)	(0.0311)	(0.2512)	(0.1272)	(0.0599)	(1.2128)	(0.1032)	(0.1024)	(0.1399)
*N*	336	28	28	336	28	28	322	28	21
Criterion function	0.2080	0.7958	0.9161	0.2277	0.8311	0.6776	0.5344	0.6453	0.6825
Hansen’s J statistic	440.928	286.474	201.538	475.804	299.181	149.073	325.954	258.123	191.088

Note: Dependent variables in logarithms. Helmert transformation applied before estimation. Heteroscedasticity and serial correlation robust standard errors between parentheses. ***, ** and ** denote significance at the 1%, 5% and 10% significance level, respectively

Estimations for all countries: 2002–2015. Estimation of GMM panel-VAR with one lag and one to four lags in the endogenous instruments. Estimations for Northern countries correspond to the period 2009–2015. Northern countries include Denmark, Finland, Norway and Sweden. Estimation of Bayesian panel-VAR. Standard errors are obtained after 100 Monte Carlo repetitions

Insufficient observations for social LTC expenditure for Southern countries

**Table 5:** Panel-VAR for medication expenditure

	LTC Expenditure (in logs)		Health LTC Expenditure (in logs)		Social LTC Expenditure (in logs)	
	All countries	Northern European Countries	All countries	Northern European Countries	All countries	Northern European Countries
*Eq: LogLTC*						
LogLTC(− 1)	0.8817***	1.7128***	0.8702***	0.9249***	2.5137***	5.1612***
	(0.0709)	(0.1086)	(0.0518)	(0.0398)	(0.4233)	(1.501)
LogMedicine(− 1)	− 0.0181*	− 0.0400***	− 0.2017	− 0.0582*	− 0.0505	− 0.0471***
	(0.0105)	(0.0099)	(0.1306)	(0.0330)	(0.2116)	(0.0108)
LogGDP pc(− 1)	0.0042	0.4970***	0.4329	0.5193***	7.9770***	1.8110
	(0.0318)	(0.0972)	(0.3535)	(0.1496)	20.013	(1.5221)
*Eq: LogMedicine*						
LogLTC(− 1)	− 0.8691**	− 1.1199***	− 0.0898***	− 0.0648***	− 0.8956***	− 1.0675***
	(0.4021)	(0.2053)	(0.0228)	(0.0092)	(0.0744)	(0.3872)
LogOutpatient(− 1)	− 0.8691**	− 1.1199***	− 0.0898***	− 0.0648***	− 0.8956***	− 1.0675***
	(0.4021)	(0.2053)	(0.0228)	(0.0092)	(0.0744)	(0.3872)
LogGDP pc(− 1)	0.2911*	5.6216***	0.1728	1.4261***	1.0177***	1.0217***
	(0.1756)	10.105	(0.1838)	(0.3744)	(0.3205)	(0.3850)
*Eq: LogGDP pc*						
LogLTC(− 1)	0.2778**	1.0973**	0.1892***	0.7529**	0.0702**	0.1744***
	(0.0940)	(0.4622)	(0.0132)	(0.1125)	(0.0358)	(0.0515)
LogMedicine(− 1)	0.0580*	0.1364***	0.0643*	0.1274***	0.0272	0.1404
	(0.0338)	(0.0365)	(0.0390)	(0.0289)	(0.0263)	(0.1479)
LogGDP pc(-1)	0.5513***	0.1283**	0.6170***	0.1488***	0.6154***	0.4630***
	(0.1169)	(0.0474)	(0.0969)	(0.0302)	(0.1331)	(0.1643)
*N*	280	28	280	28	266	28
Criterion function	220.000	40.000	200.000	40.000	50.000	30.000
Hansen’s J statistic	0.1994	0.8610	0.2324	0.7966	0.7058	0.6841

Note: Dependent variables in logarithms. H elmert transformation applied before estimation. Heteroscedasticity and serial correlation robust standard errors between parentheses. ***, ** and ** denote significance at the 1%, 5% and 10% significance level, respectively

Estimations for all countries: 2002–2015. Estimation of GMM panel-VAR with one lag and one to four lags in the endogenous instruments. Estimations for Northern countries correspond to the period 2009–2015. Northern countries include Denmark, Finland, Norway and Sweden

### Impulse response functions

An important visual examination is reported by  the impulse response functions (IRF) of the response variable (in logs) to a one standard deviation shock in an impulse variable (in logs). Each figure represents the dynamics of the response, as well as the lower and upper confidence intervals at a 95% significance level.^29^

**Fig. 1:**
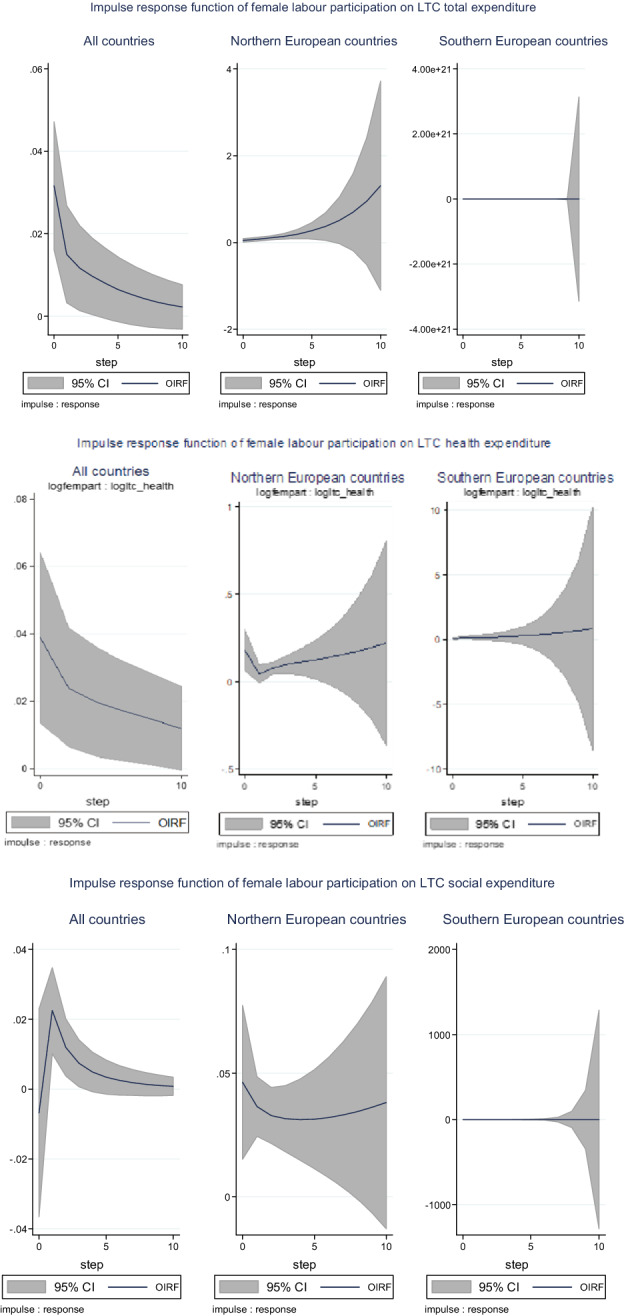
Impulse response function of female labour participation. Figures show the orthogonalized impulse response functions (OIRF) along with 95% confidence intervals (“impulse variable” in logs; “response variable” in logs) based on 200 Monte Carlo simulations with 200 repetitions. In each figure, the horizon (5 periods) is set on the x-axis and the percentage change in the response variable is on the y-axis. Estimation of GMM panel-VAR for all countries and Bayesian panel-VAR for Northern and Southern countries. Step: time unit equivalent to one year

Figure 1 plots the IRF of a one standard deviation shock in the female labour participation over LTC expenditures (total, health, and social) for all countries and for the group of Northern and Southern countries. Response functions depict the evolution of the response variable during the subsequent periods (years) resulting from a change in the impulse variable (female labour participation) by one standard deviation. We find that an increase in female labour participation in one period leads to a decrease in the total LTC expenditures in the subsequent period. When we examine the specific effects of health and social LTC expenditures, the described pattern suggests an initial increase (of 0.03% and 0.02%, respectively).

In Northern countries, we find that an increase in female labour participation gives rise to an increase in total LTC expenditures by 0.04%, which subsequently increases until it reaches 0.25% in five periods. For health LTC expenditures, we observe a V-shaped pattern with an increase of 0.2% in the first period, followed by a decrease in the next period. In contrast, in Southern countries, the effect on total and social LTC expenditures is imperceptible, and the increase in health LTC expenditures is the only item that deserves noting (0.01% in the first period, with an increasing tendency).

**Fig. 2:**
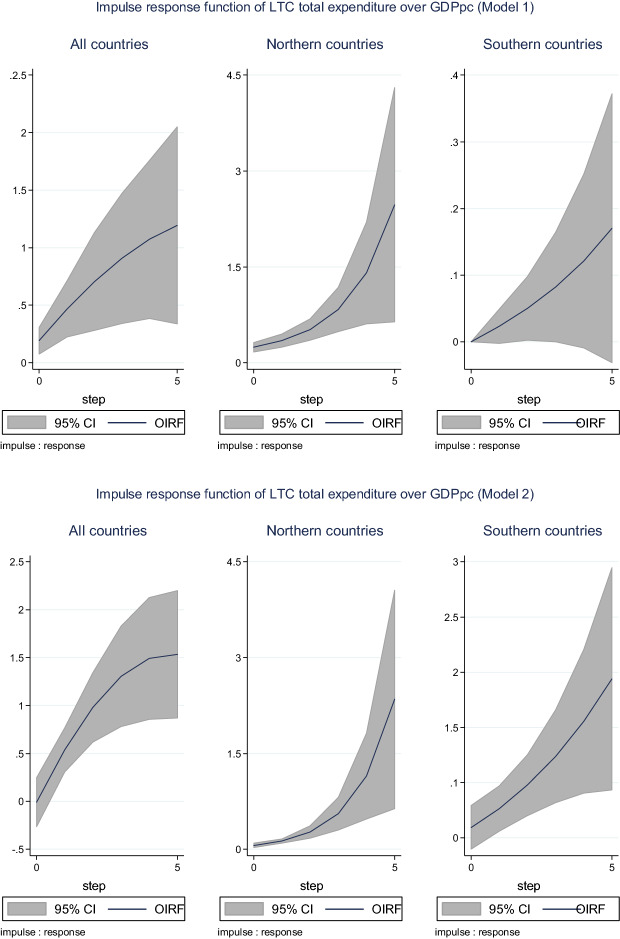
Impulse response function of LTC expenditure over GDP pc. Figures show the orthogonalized impulse response functions (OIRF) along with 95% confidence intervals (“impulse variable” ⟶ “response variable”) based on 200 Monte Carlo simulations with 200 repetitions. In each figure, the horizon (5 periods) is set on the x-axis and the percentage change in the response variable is on the y-axis. Model 1 (FemPart, LTC, GDP pc) and Model 2 (LTC, HC, GDP pc). Estimation of GMM panel-VAR for both models with one lag and one to four lags in the endogenous instruments has been estimated. Step: time unit equivalent to one year

Figure 2 compares the IRF of total LTC expenditure over GDP pc in the two models. A one standard deviation increase of total LTC expenditure increases GDP pc between 1.25% (model 1) and 1.5% (model 2) after 5 years and would reach almost 2.5% for Northern countries. GDP pc growth appears to stabilize in the last two years for the all-country sample, whereas for the Northern and Southern countries, it still shows a steep slope.

**Fig. 3:**
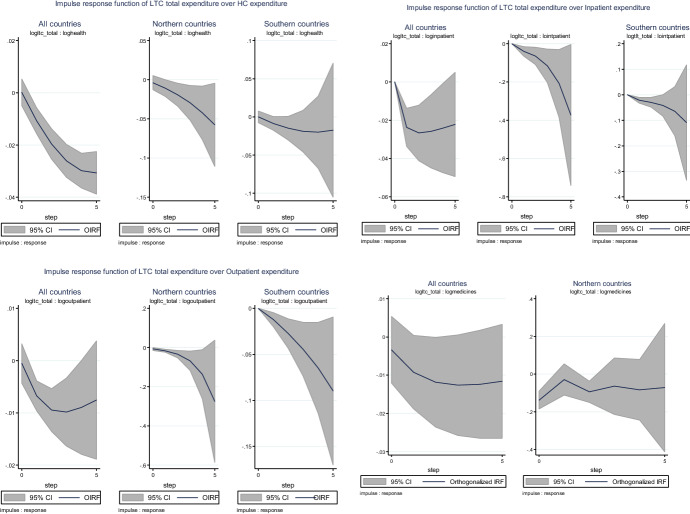
Impulse response function of LTC expenditure over HC expenditure. Figures show the orthogonalized impulse response functions (OIRF) along with 95% confidence intervals (“impulse variable” in logs; “response variable” in logs) based on 200 Monte Carlo simulations with 200 repetitions. In each figure, the horizon (5 periods) is set on the x-axis and the percentage change in the response variable is on the y-axis. Estimation of GMM panel-VAR for all countries and Bayesian panel-VAR for Northern countries. Step: time unit equivalent to one year

Figure 3 shows the IRF of total LTC expenditure over HCE and its components. One standard deviation increases of total LTC expenditure lead to a reduction in total HCE by 0.5% after five periods (− 0.5% for Northern countries and − 0.2% for Southern countries). Yet, the impact over inpatient expenditure is higher than for outpatient expenditure. Finally, a one standard deviation increase of LTC expenditure reduces medicines expenditure by almost 0.2% in Northern countries, even though the overall impact is roughly − 0.1%.

### Robustness checks

#### Alternative Granger causality tests

As an additional approach to gauge the predictive power of the variables in the baseline panel-VAR model, in addition to the Granger causality tests in line with Abrigo and Love (2016), we also have performed a sequence of pairwise Dumistrescu and Hurlin (2012) Granger causality tests for all model variables. This test is developed for heterogeneous panels based on individual Wald statistics of Granger non-causality computed for each cross section unit and then averaged over all cross section units in the sample.^30^

#### Unit root tests

We estimate evidence of panel stationarity. This result adds to the long debate on the stationarity of GDP per capita.^31^ Finally, as a robustness test, we have performed the test proposed by the Carrión-Silvestre et al. (2005), which has the advantage of allowing multiple structural breaks in panel data. The results (available upon request) strongly reject the null hypotheses of unit roots and as the test statistics is based on the use of bootstrap critical values, it is robust to the presence of cross section dependence.

## Conclusion

This paper has conducted the first large-scale cross-country analysis of dynamic determinants of long term care ( LTC ) spending, and its impact on health care and economic performance (GDP pc). Our estimates draw on a panel-VAR (Abrigo and Love 2016) approach that exploits rich longitudinal evidence from a panel for 25 industrialised countries from 2002 to 2015.

We have empirically examined the effect of dynamic changes in the labour market participation of traditional caregivers (women over 40 years of age) on LTC expenditures,  alongside the dynamic effect of LTC spending on health expenditures and GDP per capita to document the presence of ‘caregiving spillovers’ on the health system and the economy. Our estimates may be affected by measurement error, as they rely on the effect of unanticipated shocks to female labour participation, healthcare and LTC expenditure. Alternatively, one could consider health or disability shocks.

Our findings suggest that a 1% increase in the labour market participation of the traditional caregiver (women over the age of 40) leads to a 1.48% increase in LTC expenditure yet reveal significant heterogeneity in its effect size among countries (reaching a maximum of 3.96% in Northern European countries, but no effect in Southern European countries). This effect is explained by the differential availability of both care and cash subsidies in Southern European countries (Spasova et al. 2018). A one percentage point increase in  female labour participation leads to an increase in LTC expenditures of 0.03% (which reaches 0.25% in Nordic European countries).

Our results are especially relevant in the context of De Henau and Himmelweit (2021) who document that in the post-Covid 19 environment, one would need to more than double the supply of care in the EU-28 and the US. Spending in LTC can give rise to a stronger caring economy, generating employment not only in the care sector, but also in related industries, which can in turn stimulate economic performance through the spending generated by new workforce. In the short term, such a government expansion of LTC spending may be partially absorbed by higher tax revenues.

Consistently  with the ‘caregiving spillover hypothesis’ put forward in this paper, we find that a 1% increase in LTC expenditures gives rise to a reduction in HCE by 0.6% in the subsequent year. Although the effect is slightly smaller in Southern European countries (0.55%), we estimate it to be  three times as large in the sample of Northern  European countries (1.78%). These estimates are explained by the fact that in Northern European countries, the provision of health and LTC is the responsibility of municipal governments, and there is a well developed system (and a pre-existing culture) of  formal caregiving (Iversen et al. 2016). Yet, the effects differ depending on the type of both health and LTC spending examined.

Finally, our results document that spending in public LTC increases GDP pc; the latter results from the effects of training formal caregivers and expanding employment in the sector.

## Supplementary Information

Below is the link to the electronic supplementary material.

Supplementary file1 (DOCX 424 kb)

## Footnotes

1 Furthermore, we consider a Granger causality analysis to assist in the identification of the direction of the link between each pair of variables, and the estimation of impulse response functions which are suitable to illustrate how the response of each endogenous variable differs according to the nature of shocks affecting them.

2 Unlike other mean-based estimators (OLS, pooled ordinary least squares, fully modified ordinary least squares, dynamic ordinary least squares, vector error-correction model), the panel-VAR estimator gives both mean-based and trend findings, allowing the series’ behavior to be monitored (Love and Zicchino 2006).

3 A public subsidy for formal LTC increases the demand for it, even when an informal caregiver is available (Coughlin et al. 1992).

4 As a share of GDP, LTC spending varies from 3.7% in the Netherlands (which relies heavily on institutional care), and 0.2% in Portugal (which relies mainly on informal care by family members), with the OECD average at 1.5% of GDP.

5 This magnitude is approximately three times higher than that observed in acute health care expenditures (Costa-Font et al. 2011).

6 The availability of a spouse caregiver, measured by male-to-female ratio among the elderly, is associated with a $28,840 (1995 U.S. dollars) annual reduction in formal LTC expenditure per additional elderly male (Yoo et al. 2004).

7 Holland et al. (2014) compare data of LTC beneficiaries and non-beneficiaries residing in California at the end of their lives. They find that LTC claimants experience significantly lower health care costs at end of life, including 14% lower total medical costs, 13% lower pharmacy costs, 35% lower inpatient admission costs, and 16% lower outpatient visit costs. Feng et al. (2020) examined the effect of LTC on hospital utilization and expenditures among the elderly in China. LTC significantly reduced the length of stay by 41%, inpatient expenditures by 17.7% and outpatient visits by 8.1%. Additionally, Rapp et al. (2015) report that the extension of LTC subsidies lowers the use of emergency care, and Costa-Font et al. (2018) find an 8% reduction in hospitalisation after the insurance extension to finance LTC.

8 Indeed, De Henau et al. (2016) estimate that the contribution to employment of an investment in the care economy (equivalent to 2% of GDP) is higher than a similar investment in construction.

9 Although previous studies have used a single-equation method that relies on a cointegrated relationship, the main assumption of cointegration is the independence of the error term, which is likely violated in the present context.

10 These problems will be addressed using recently developed estimation techniques for unit roots (Im-Pesaran-Shin test) and Dumitrescu-Hurlin causality test for heterogeneous panels.

11 We use impulse response function and forecast error variance decomposition (FEVD) tests to find information transmission in a dynamic panel analysis, and specifically to understand the responsiveness of healthcare expenditure and GDP to shocks in LTC.

12 Monetary variables are expressed in constant prices, constant PPPs, 2010 US Dollars.

13 Wang (2015) analyses data for 34 OCDE countries and finds that appropriate spending on healthcare improves human capital and contributes to economic growth through higher productivity. Erdil and Yetkiner (2009) examine the effect of HCE and per capita (GDPpc) in a sample of 75 countries that ranged from low to high income.

14 Forward means differencing can be considered as a combination of first differences and generalised least squares (Arellano and Bover 1995).

15 Its null hypothesis is that the included instruments are valid in the sense that they are uncorrelated with the error term. Simultaneously, the excluded instruments are properly excluded. A fundamental issue in the estimation of a panel-VAR model is to determine the optimal lag order ($p$). A higher number of lags tends to provide more efficient estimates because they ensure that ${\mathrm{E}[\upvarepsilon }_{\mathrm{i},\mathrm{t}}]=0$; however, at the same time, as the number of lags increases, fewer observations are available to calculate the model.

16 The stability condition of the panel-VAR model requires that the unit of all roots of matrix *A* are within the unit circle. This condition guarantees the invertibility of the model and allows it to be expressed as an infinite-order moving average of the innovations (${\varepsilon }_{i,t}$).

17 We assume that variables that first appear in the system must be more exogenous, and that those appearing afterwards are more endogenous, such that the variables appearing first affect the others simultaneously and with lags, whereas the variables that appear at the end, only affect the ones before throughout the lags. The enforcement of these restrictions allows uncorrelated residuals to be obtained, which are known as Orthogonalised Impulse Response Functions (OIRF). The OIRF serves as the basis to obtain a variance decomposition of the forecast error, which indicates the relative importance of the variation of a variable when faced with a shock in another, holding all other shocks constant at zero.

18 Australia, Austria, Belgium, Canada, Czech Republic, Denmark, Estonia, Finland, France, Germany, Greece, Hungary, Iceland, Italy, Korea, Latvia, Luxembourg, Netherlands, Norway, Poland, Portugal, Slovak Republic, Slovenia, Spain, Sweden, Switzerland, and United States.

19 The underlying intuition about the estimation of the models for the two groups of European countries is to observe whether significant differences exist, given the fact that the countries that form part of each group have important similarities (cultural values, consideration of women’s role, consideration of family, or whether the state is responsible for the caregiving of dependent people).

20 The definition of health-care expenditure and long-term care expenditure, as well as their components, follows the System of Health Accounts methodology. Statistical information with this methodology is highly reliable and reputable and has been widely used. For example, data on health and long-term care expenditure have been used to evaluate health care system performance and its impact on the living conditions of EU citizens. https://ec.europa.eu/economy_finance/publications/occasional_paper/2010/pdf/ocp74_en.pdf. Nevertheless, the reports OECD (2017, 2018) recognize that separate guidelines may be important for national analysis in lower-income countries. However, we are not concerned by this heterogeneity because the group of low-income countries is not included in our sample.

21 Medical or health-related LTC expenditure per capita: includes medical or nursing care (e.g. wound dressing, administering medication, health counselling, palliative care, pain relief and medical diagnosis with relation to a LTC condition), preventive activities to avoid deterioration in long-term health conditions and personal care services which provide help with activities of daily living (ADL) such as eating (support with food intake), bathing, washing, dressing, getting in and out of bed, getting to and from the toilet and managing incontinence.

22 Social LTC expenditure per capita, which consists of assistance services that enable a person to live independently. They relate to help with instrumental activities of daily living (IADL) such as shopping, laundry, cooking, performing housework, managing finances, and using the telephone.

23 Which includes curative care (inpatient and outpatient), rehabilitative care, preventive care and consumption of medical goods.

24 Analysis of long-term care spending performed by Mueller et al. (2020) suggests a close cross-country alignment of cost items for inpatient long-term care and there is a high consensus that costs for the frail elderly and elderly with dementia living in institutions are included under long-term (health) care. However, there are some differences across countries in the consideration of medicines consumed in residential facilities. Furthermore, there has been identified a group of countries that have under-reported long-term care spending elements (Australia, Denmark, Greece, Iceland, Poland, the Slovak Republic, and the United States).

25 The presence of unit roots could give rise to a spurious association, indicating persistence in response to innovations when, in fact, there is none.

26 However, before considering them and as a starting point, a specification has been sought to satisfy the test of overidentification proposed by Hansen (1982), keeping in mind that this is an indispensable requirement before searching for the lag length that minimises MMSCAIC, MMSCBIC, and MMSCHQIC.

27 Regarding GDPpc, we find that it can explain up to 34.3% of the FEV of the total LTC expenditures in Southern countries, which is three times higher than in Nordic countries and ten times higher than in the group of all countries. In contrast, total LTC expenditures can explain 13.44% of the FEV of GDPpc in Nordic countries, considerably different from the other cases (5.73% for all countries and 3.04% for Southern countries).

28 We find a small effect for Northern and Southern countries (0.82% and 0.30%, respectively).

29 Given that the IRFs are computed using the estimated panel-VAR coefficients, the standard errors of these coefficients are retrieved from Monte Carlo simulations, in which the parameters of the model are re-calculated 200 times using the estimated coefficients and their variance–covariance matrices as underlying distribution.

30 The null hypothesis states that there is no causal relationship for any of the units of the panel, whereas under the alternative hypothesis there is a causality relationship for at least one cross-sectional unit.

31 While Cheung and Chinn (1996), Rapach (2002), and Ozturk et al. (2008) have obtained results against the stationarity of GDP for several OECD panels, Fleissig and Strauss (1999) and Chang et al. (2009) have obtained results in favour of the hypothesis of stationarity.
