# Supplementary material for: ‘Investing’ in care for old age? An examination of long-term care expenditure dynamics and its spillovers
Source: Empir Econ. 2022 May 27;64(1):1–30. doi: 10.1007/s00181-022-02246-0 (PMC9137442; doi:10.1007/s00181-022-02246-0)
Supplement: Supplementary file 1 — Supplementary file1 (DOCX 424 kb) [file 181_2022_2246_MOESM1_ESM.docx]

**Appendix for Online Publication**

**Appendix A**

**Figure A1. Relationship between per capita total LTC expenditure and per capita GDP (constant prices, constant PPPs, OECD base year).**

|  |
| --- |

Notes LTC expenditure per capita is the sum of social and health LTC expenditure per capita.

Per capita total LTC expenditure in constant prices, constant PPPs, 2010 US Dollars. AUSTL: Australia; AUSTI: Austria; BEL: Belgium; CAN: Canada; CZE: Czech Republic; DEN: Denmark; EST: Estonia; FIN: Finland; FRA: France; GER: Germany; GRE: Greece; HUN: Hungary; ICE: Iceland; IRE: Ireland; ITA: Italy; KOR: Korea; LAT: Latvia; LUX: Luxembourg; NET: Netherlands; NOR: Norway; POL: Poland; POR: Portugal; SLR: Slovak Republic; SLO: Slovenia; SP: Spain; SWE: Sweden; SWI: Switzerland; US: United States. Blue line corresponds to the prediction for per capita total LTC expenditure from estimation of a fractional polynomial of percentage of per capita GDP and grey lines correspond to confidence intervals at 95% level of significance. Own work using data from OECD Stats.

**Figure A2. Relationship between per capita total LTC expenditure and female labour participation**

|  |
| --- |

Notes: LTC expenditure per capita is the sum of social and health LTC expenditure per capita.

Per capita total LTC expenditure in constant prices, constant PPPs, 2010 US Dollars. Blue line corresponds to the prediction for per capita total LTC expenditure from estimation of a fractional polynomial of percentage of female labour participation (40 years and older) and grey lines correspond to confidence intervals at 95% level of significance. Own work using data from OECD Stats.

**Figure A3. Relationship between per capita total LTC expenditure (and per capita total HC expenditure (constant prices, constant PPPs, OECD base year).**

|  |
| --- |

Notes: LTC expenditure per capita is the sum of social and health LTC expenditure per capita.

Healthcare expenditure per capita does not include LTC expenditure related to health services.

Variables in constant prices, constant PPPs, 2010 US Dollars. Blue line corresponds to the prediction for LTC expenditure from estimation of a fractional polynomial of percentage of HC expenditure and grey lines correspond to confidence intervals at 95% level of significance. Own work using data from OECD Stats.

**Table A1. Definition of the variables**

| Name | Definition |
| --- | --- |
| LTC Total | Per capita total LTC expenditure (constant prices, constant PPPs, 2010 US Dollars) |
| LTC Health | Per capita health LTC expenditure (constant prices, constant PPPs, 2010 US Dollars) |
| LTC Social | Per capita social LTC expenditure (constant prices, constant PPPs, 2010 US Dollars) |
| HC total | Per capita total healthcare expenditure (constant prices, constant PPPs, 2010 US Dollars).  It does not include health LTC expenditure |
| Inpatient | Per capita expenditure in inpatient care (constant prices, constant PPPs, 2010 US Dollars) |
| Outpatient | Per capita expenditure in outpatient care (constant prices, constant PPPs, 2010 US Dollars). |
| Medicines | Per capita expenditure in prescribed medicines (constant prices, constant PPPs, 2010 US Dollars) |
| FemPart | Female labour participation (age 40 and older) |
| GDP pc | Per capita gross domestic product (constant prices, constant PPPs, 2010 US 1,000 Dollars) |

**Table A2. Country availability of information**

|  | HC Total | Inpatient | Outpatient | Medicines | LTC  Total | LTC Health | LTC Social | FemPart |
| --- | --- | --- | --- | --- | --- | --- | --- | --- |
| Australia | X | X | X | X | X | X | X | X |
| Austria | X | X | X | X | X | X | X | X |
| Belgium | X | X | X | X | X | X | X | X |
| Canada | X | X | X | X | X | X | NO | X |
| Czech Rep. | X | X | X | X | X | X | X | X |
| Denmark | X | X | X | X | X | X | X | X |
| Estonia | X | X | X | X | X | X | X | X |
| Finland | X | X | X | X | X | X | X | X |
| France | X | X | X | X | X | X | X | X |
| Germany | X | X | X | X | X | X | X | X |
| Greece | 2009-2015 | 2009-2015 | 2009-2015 | NO | 2009-2015 | 2009-2015 | NO | X |
| Hungary | X | X | X | X | X | X | X | X |
| Iceland | X | X | X | X | X | X | X | X |
| Italy | 2009-2015 | NO | X | NO | 2009-2015 | 2009-2015 | X | X |
| Korea | X | X | X | X | X | X | X | X |
| Latvia | X | X | X | X | X | X | X | X |
| Luxembourg | X | X | X | X | X | X | X | X |
| Netherlands | X | X | X | NO | X | X | X | X |
| Norway | X | X | X | X | X | X | X | X |
| Poland | X | X | X | X | X | X | X | X |
| Portugal | X | X | X | NO | X | X | X | X |
| Slovak Rep. | X | X | X | X | X | X | X | X |
| Slovenia | X | X | X | X | X | X | X | X |
| Spain | X | X | X | X | X | X | X | X |
| Sweden | X | X | X | X | X | X | X | X |
| Switzerland | X | X | X | X | X | X | X | X |
| United States | X | NO | NO | X | X | X | X | X |
| N (2002-2015) | 350 | 336 | 350 | 322 | 350 | 350 | 350 | 378 |
| Southern countries | 28 | 21 | 28 | 7 | 28 | 28 | 21 | 28 |
| Northern countries | 28 | 28 | 28 | 28 | 28 | 28 | 28 | 28 |

X: means information available for the whole period 2002-2015.

NO: means not available for the period 2002-2015.

Southern countries (Greece, Italy, Portugal, Spain): observations for the period 2009-2015.

Northern countries (Denmark, Finland, Norway, Sweden): observations for the period 2009-2015.

Italy and Greece will not be included in the analysis for the period 2002-2015, but will be used in a posterior analysis for the subperiod 2009-2015.

**Table A3. Descriptive statistics**

|  | GDP pc | LTC Total | LTC Health | LTC Social | HC Total | Inpatient | Outpatient | Medicines | FemPart (%) |
| --- | --- | --- | --- | --- | --- | --- | --- | --- | --- |
| Australia | 40.58 | 313.36 | 38.37 | 275.00 | 2,401.57 | 901.20 | 794.60 | 289.59 | 67.48 |
|  | 2.13 | 142.67 | 32.77 | 114.97 | 213.62 | 61.68 | 86.51 | 12.32 | 2.73 |
| Austria | 41.56 | 743.06 | 452.29 | 290.78 | 2,617.29 | 1,201.76 | 716.76 | 365.66 | 63.49 |
|  | 1.75 | 118.06 | 47.24 | 71.73 | 174.89 | 89.81 | 49.16 | 14.62 | 4.59 |
| Belgium | 39.50 | 917.37 | 716.56 | 200.81 | 2,181.07 | 828.19 | 523.23 | 374.81 | 57.89 |
|  | 1.30 | 177.08 | 132.46 | 50.17 | 194.59 | 82.00 | 41.36 | 32.61 | 4.70 |
| Canada | 40.50 | 466.79 | 466.79 | - | 2,286.93 | 612.13 | 753.78 | 261.42 | 72.45 |
|  | 1.42 | 21.37 | 21.37 | - | 232.11 | 48.69 | 129.55 | 20.11 | 1.49 |
| Czech Rep. | 26.63 | 175.37 | 106.11 | 69.26 | 1,434.57 | 513.35 | 402.86 | 248.94 | 68.72 |
|  | 2.44 | 82.70 | 84.77 | 17.52 | 135.27 | 48.91 | 81.65 | 30.07 | 2.59 |
| Denmark | 43.52 | 2,101.19 | 927.07 | 1,174.14 | 2,617.36 | 1,088.04 | 927.34 | 165.71 | 74.94 |
|  | 1.19 | 106.85 | 84.22 | 38.82 | 187.50 | 77.97 | 109.00 | 23.42 | 1.08 |
| Estonia | 22.72 | 101.71 | 39.11 | 62.59 | 940.57 | 340.31 | 247.24 | 123.44 | 78.41 |
|  | 2.93 | 34.92 | 16.92 | 18.82 | 181.92 | 52.87 | 65.72 | 15.71 | 2.39 |
| Finland | 38.38 | 905.22 | 484.39 | 420.84 | 1,968.21 | 755.99 | 694.86 | 245.04 | 77.10 |
|  | 1.74 | 187.15 | 77.48 | 127.90 | 193.68 | 52.12 | 111.41 | 16.55 | 1.37 |
| France | 35.95 | 523.21 | 401.93 | 121.29 | 2,604.14 | 1,072.36 | 439.21 | 415.58 | 67.20 |
|  | 0.84 | 94.58 | 72.92 | 23.02 | 106.68 | 25.07 | 37.99 | 13.31 | 2.01 |
| Germany | 39.50 | 610.79 | 424.20 | 186.59 | 2,919.36 | 1,063.86 | 722.42 | 481.20 | 71.23 |
|  | 2.26 | 92.79 | 59.44 | 33.42 | 394.28 | 131.23 | 112.47 | 63.67 | 4.69 |
| Greece | 27.33 | 14.87 | 14.87 | - | 1,440.71 | 654.99 | 178.13 | 473.33 | 52.02 |
|  | 2.97 | 5.47 | 5.47 | - | 343.01 | 168.15 | 25.23 | 167.01 | 3.68 |
| Hungary | 21.76 | 164.66 | 51.87 | 112.79 | 1,057.43 | 376.74 | 195.32 | 251.67 | 59.10 |
|  | 1.38 | 22.68 | 9.31 | 16.16 | 67.88 | 23.04 | 12.27 | 32.60 | 3.52 |
| Iceland | 40.50 | 1,192.54 | 675.91 | 516.65 | 2,179.14 | 965.78 | 569.71 | 219.05 | 85.78 |
|  | 2.68 | 221.65 | 64.95 | 213.11 | 123.70 | 144.99 | 41.14 | 31.75 | 1.29 |
| Italy | 35.04 | 277.36 | 235.49 | 40.62 | 2,097.43 | 835.18 | 672.47 | - | 49.36 |
|  | 1.50 | 10.33 | 3.43 | 5.16 | 93.94 | 37.40 | 184.02 | - | 4.81 |
| Korea | 28.74 | 137.03 | 116.44 | 20.63 | 877.79 | 253.54 | 277.70 | 218.45 | 59.61 |
|  | 3.68 | 90.20 | 100.90 | 16.36 | 178.93 | 41.81 | 61.47 | 44.45 | 1.63 |
| Latvia | 18.41 | 107.74 | 46.37 | 61.07 | 592.55 | 233.73 | 99.79 | 88.68 | 74.15 |
|  | 2.96 | 21.16 | 8.37 | 16.77 | 73.36 | 34.46 | 13.55 | 23.32 | 3.45 |
| Luxembourg | 84.52 | 1,793.53 | 950.78 | 842.74 | 4,005.21 | 1,414.35 | 1,372.01 | 439.44 | 56.99 |
|  | 3.32 | 213.95 | 101.61 | 118.64 | 340.27 | 66.51 | 299.46 | 71.87 | 5.68 |
| Netherlands | 44.31 | 1,299.14 | 927.60 | 371.54 | 2,424.64 | 806.26 | 738.50 | - | 64.80 |
|  | 1.69 | 335.80 | 281.66 | 57.54 | 411.37 | 58.59 | 203.47 | - | 5.23 |
| Norway | 58.29 | 2,377.96 | 1,251.01 | 1,126.97 | 3,032.43 | 1,390.81 | 672.42 | 221.54 | 77.51 |
|  | 1.50 | 248.67 | 179.14 | 84.91 | 235.98 | 117.19 | 120.85 | 29.09 | 1.13 |
| Poland | 19.57 | 100.90 | 70.29 | 30.63 | 786.00 | 378.04 | 156.43 | 110.92 | 57.49 |
|  | 3.09 | 25.92 | 14.18 | 15.61 | 158.94 | 90.68 | 30.24 | 9.73 | 2.01 |
| Portugal | 26.67 | 29.42 | 28.70 | 0.71 | 1,671.43 | 441.47 | 590.59 | - | 67.01 |
|  | 0.61 | 11.82 | 11.77 | 0.33 | 110.10 | 59.99 | 49.74 | - | 2.33 |
| Slovak Rep. | 23.20 | 123.84 | 6.52 | 116.69 | 1,243.07 | 369.54 | 280.06 | 359.96 | 66.59 |
|  | 3.70 | 12.34 | 0.51 | 16.11 | 235.32 | 66.46 | 101.33 | 25.98 | 2.17 |
| Slovenia | 26.99 | 279.17 | 207.98 | 71.19 | 1,410.36 | 567.31 | 369.09 | 230.66 | 62.73 |
|  | 1.83 | 46.02 | 29.31 | 19.56 | 102.57 | 49.40 | 49.14 | 15.60 | 2.82 |
| Spain | 31.79 | 350.14 | 178.07 | 172.06 | 1,744.36 | 571.92 | 518.41 | 344.51 | 58.14 |
|  | 1.10 | 110.02 | 52.25 | 61.49 | 149.02 | 65.58 | 41.96 | 31.62 | 8.00 |
| Sweden | 41.06 | 2,041.32 | 568.84 | 1,472.48 | 2,599.14 | 931.05 | 957.96 | 255.53 | 81.62 |
|  | 2.14 | 550.29 | 442.42 | 124.59 | 172.35 | 34.71 | 90.22 | 17.39 | 1.67 |
| Switzerland | 51.88 | 1,064.80 | 701.33 | 363.46 | 2,780.07 | 1,256.32 | 847.98 | 401.28 | 74.75 |
|  | 2.41 | 118.11 | 87.46 | 33.98 | 250.13 | 76.12 | 65.43 | 14.07 | 2.03 |
| United States | 48.74 | 264.68 | 260.01 | 4.68 | 3,733.36 | - | - | 262.88 | 69.71 |
|  | 1.88 | 17.70 | 17.70 | 0.28 | 1,311.85 | - | - | 59.41 | 0.96 |
| All countries | 36.95 | 705.04 | 393.22 | 300.97 | 2,072.20 | 750.89 | 567.56 | 271.92 | 67.27 |
|  | 13.85 | 710.22 | 368.89 | 397.07 | 928.13 | 356.48 | 310.66 | 109.88 | 9.58 |

Own work using OECE Stats. Standard deviations in italics.

LTC and HC expenditures are expressed in per capita terms (constant prices, constant PPPs, 2010 US Dollars)

GDP pc is expressed in per capita terms (constant prices, 1,000 constant PPPs, 2010 US Dollars)

**Appendix B**

**Table B1. Panel unit root tests. All countries**

|  | Harris-Tzavalis test | | Im-Pesaran-Shin test | |
| --- | --- | --- | --- | --- |
|  | Statistic | p-value | Statistic | p-value |
| Log LTC_Total | 0.2488 | 0.0387 | -23.739 | 0.0088 |
| Log LTC_Health | 0.1883 | 0.0452 | -26.802 | 0.0037 |
| Log LTC_Social | 0.6873 | 0.0439 | -16.857 | 0.0459 |
| Log HC_Total | -0.4640 | 0.0474 | -20.708 | 0.0192 |
| Log Inpatient | 0.2501 | 0.0467 | -24.657 | 0.0068 |
| Log Outpatient | 0.1714 | 0.0332 | -36.992 | 0.0001 |
| Log Medicines | 0.1345 | 0.0013 | -24.660 | 0.0068 |
| Log GDP pc | -0.7289 | 0.0001 | -19.936 | 0.0231 |

Harris-Tzavalis unit-root test (1999): Ho: Panels contain unit roots vs. Ha: Panels are stationary.

Im-Pesaran-Shin unit-root test (2003): Ho: All panels contain unit roots vs. Ha: Some panels are stationary.

**Table B2. Panel-VAR model selection. All countries**

|  | CD | J statistic | J pvalue | MMSCBIC | MMSCAIC | MMSCHQIC |
| --- | --- | --- | --- | --- | --- | --- |
| Panel-VAR Model 1: Log(Femp_Part), , log(LTC_total), Log(GDP pc) | | | | | | |
| Lag 1 | 0.9999569 | 12.92761 | 0.3743241 | **-52.27705** | **-11.07239** | **-27.69537** |
| Lag 2 | 0.9999584 | 6.038905 | 0.6428738 | -37.43087 | -9.961095 | -21.04308 |
| Lag 3 | 0.9999367 | 4.513766 | 0.3409184 | -17.22112 | -3.486234 | -9.027229 |
| Panel-VAR Model 1: Log(Femp_Part), , log(LTC_health), Log(GDP pc) | | | | | | |
| Lag 1 | 0.999929 | 13.94894 | 0.3039807 | **-51.25572** | **-10.05106** | **-26.67404** |
| Lag 2 | 0.9999281 | 6.441629 | 0.5978891 | -37.02815 | -9.558371 | -20.64036 |
| Lag 3 | 0.9998983 | 2.420629 | 0.6589026 | -19.31426 | -5.579371 | -11.12037 |
| Panel-VAR Model 1: Log(Femp_Part), , log(LTC_social), Log(GDP pc) | | | | | | |
| Lag 1 | 0.9998795 | 7.527316 | 0.8208933 | **-57.30481** | **-16.47268** | **-32.95816** |
| Lag 2 | 0.9998954 | 4.105701 | 0.8474629 | -39.11572 | -11.89439 | -22.88461 |
| Lag 3 | 0.9998777 | 0.6963365 | 0.9517799 | -20.91437 | -7.303663 | -12.79882 |
| Panel-VAR Model 2: log(LTC_total), Log(HC_total), Log(GDP pc) | | | | | | |
| Lag 1 | 0.9998333 | 13.32586 | 0.3458075 | **-51.87881** | **-10.67414** | **-27.29713** |
| Lag 2 | 0.9998853 | 10.27741 | 0.2460933 | -33.19237 | -5.722599 | -16.80459 |
| Lag 3 | 0.9998262 | 8.675323 | 0.0697475 | -13.05957 | 0.675322 | -4.865672 |
| Panel-VAR Model 2: log(LTC_total), Log(Outpatient), Log(GDP pc) | | | | | | |
| Lag 1 | 0.9998612 | 9.443865 | 0.6646233 | **-54.83517** | **-14.55613** | **-30.83598** |
| Lag 2 | 0.9998633 | 6.780511 | 0.5604898 | -36.07219 | -9.21959 | -20.07273 |
| Lag 3 | 0.9998247 | 3.482858 | 0.4804895 | -17.94349 | -4.51714 | -9.943757 |
| Panel-VAR Model 2: log(LTC_total), Log(Inpatient), Log(GDP pc) | | | | | | |
| Lag 1 | 0.9998811 | 10.10867 | 0.6064276 | **-54.05662** | **-13.89133** | **-30.12867** |
| Lag 2 | 0.9998823 | 10.66469 | 0.2214318 | -32.11217 | -5.335315 | -16.16021 |
| Lag 3 | 0.9998237 | 4.807544 | 0.3076206 | -16.58089 | -3.192456 | -8.604902 |
| Panel-VAR Model 2: log(LTC_total), Log(Medicines), Log(GDP pc) | | | | | | |
| Lag 1 | 0.9998857 | 9.686755 | 0.8091055 | **-54.76133** | **-16.31325** | **-31.89954** |
| Lag 2 | 0.9998817 | 8.565531 | 0.3802608 | -33.06652 | -7.434469 | -17.82533 |
| Lag 3 | 0.9998465 | 2.846512 | 0.5838309 | -17.96951 | -5.153480 | -10.34892 |
| Panel-VAR Model 2: log(LTC_health), Log(HC_total), Log(GDP pc) | | | | | | |
| Lag 1 | 0.999793 | 24.03128 | 0.6201427 | **-41.17339** | **-2.131275** | **-16.59171** |
| Lag 2 | 0.9997873 | 15.86605 | 0.0443379 | -27.60372 | -1.0339467 | -11.21594 |
| Lag 3 | 0.9997478 | 8.367966 | 0.078992 | -13.36692 | -0.367966 | -5.173029 |
| Panel-VAR Model 2: log(LTC_health), Log(Outpatient), Log(GDP pc) | | | | | | |
| Lag 1 | 0.9997352 | 9.976144 | 0.6180536 | **-54.30289** | **-14.02386** | **-30.30378** |
| Lag 2 | 0.9997514 | 6.412948 | 0.6010789 | -36.43974 | -9.587052 | -20.44028 |
| Lag 3 | 0.9997364 | 1.413159 | 0.8419047 | -20.01319 | -6.586841 | -12.01346 |
| Panel-VAR Model 2: log(LTC_health), Log(Inpatient), Log(GDP pc) | | | | | | |
| Lag 1 | 0.9997637 | 11.03594 | 0.5258408 | **-53.12935** | **-12.96406** | **-29.20139** |
| Lag 2 | 0.999766 | 7.882184 | 0.4450627 | -34.89468 | -8.117816 | -18.94271 |
| Lag 3 | 0.9997762 | 3.110056 | 0.5395797 | -18.27837 | -4.889944 | -10.30239 |
| Panel-VAR Model 2: log(LTC_health), Log(Medicines), Log(GDP pc) | | | | | | |
| Lag 1 | 0.999809 | 13.31256 | 0.3467381 | **-49.13552** | **-10.68744** | **-26.27374** |
| Lag 2 | 0.9997997 | 8.979212 | 0.3440526 | -32.65284 | -7.020788 | -17.41165 |
| Lag 3 | 0.9997517 | 1.268108 | 0.8667628 | -19.54792 | -6.731892 | -11.92732 |
| Panel-VAR Model 2: log(LTC_social), Log(HC_total), Log(GDP pc) | | | | | | |
| Lag 1 | 0.9998694 | 17.49857 | 0.1317853 | **-46.94908** | **-8.501426** | **-22.84415** |
| Lag 2 | 0.9998593 | 7.418323 | 0.4922428 | -35.54678 | -6.581677 | -19.47682 |
| Lag 3 | 0.9998529 | 6.517208 | 0.1637093 | -14.96534 | -1.482792 | -6.930365 |
| Panel-VAR Model 2: log(LTC_social), Log(Outpatient), Log(GDP pc) | | | | | | |
| Lag 1 | 0.9996643 | 4.766925 | 0.9653141 | **-59.10919** | **-19.23307** | **-35.36201** |
| Lag 2 | 0.9997017 | 1.749843 | 0.9877441 | -40.83424 | -14.25016 | -25.00278 |
| Lag 3 | 0.9996984 | 0.439831 | 0.9790878 | -20.85221 | -7.560168 | -12.93648 |
| Panel-VAR Model 2: log(LTC_social), Log(Inpatient), Log(GDP pc) | | | | | | |
| Lag 1 | 0.9998296 | 6.563949 | 0.885035 | **-56.77343** | **-17.43605** | **-33.36171** |
| Lag 2 | 0.9996549 | 3.904682 | 0.865619 | -38.32024 | -12.09532 | -22.71242 |
| Lag 3 | 0.9991048 | 1.795036 | 0.773390 | -19.31742 | -6.204964 | -11.51352 |
| Panel-VAR Model 2: log(Medicines), Log(HC_total), Log(GDP pc) | | | | | | |
| Lag 1 | 0.9997845 | 2.590712 | 0.997808 | **-59.17922** | **-21.40929** | **-36.73353** |
| Lag 2 | 0.999743 | 5.224027 | 0.733385 | -35.95593 | -10.77597 | -20.99213 |
| Lag 3 | 0.9995951 | 1.013102 | 0.907802 | -19.57688 | -6.986898 | -12.09498 |

CD: coefficient of determination; J statistic: Hansen’s (1982) J statistic; J pvalue: p-value for the Hansen’s (1982) J statistic;

MMSCAIC: moment and model selection criteria (MMSC) proposed by Andrews and Lu (1002) based on AIC (Akaike information criteria).

MMSCBIC: moment and model selection criteria (MMSC) proposed by Andrews and Lu (1002) based on BIC (Bayesian information criteria).

MMSCHQIC: moment and model selection criteria (MMSC) proposed by Andrews and Lu (1002) based on HQIC (Hannan-Quinn information criteria). GMM panel-VAR for both models with one to four lags in the endogenous instruments has been estimated

**Table B3. Granger causality tests. All countries**

|  | Chi2 | df | pvalue |  | Chi2 | df | pvalue |  | Chi2 | df | pvalue |
| --- | --- | --- | --- | --- | --- | --- | --- | --- | --- | --- | --- |
| **Panel-VAR Log(Fem_part), Log(LTC_total), Log(GDP pc)** | | | | **Panel-VAR Log(Fem_part), Log(LTC_health), Log(GDP pc)** | | | | **Panel-VAR Log(Fem_part), Log(LTC_social), Log(GDP pc)** | | | |
| Eq: Log(Femp_part) |  |  |  | Eq: Log(Femp_part) |  |  |  | Eq: Log(Femp_part) |  |  |  |
| Log(LTC_total) | 1.744 | 1 | 0.1870 | Log(LTC_health) | 1.827 | 1 | 0.1760 | Log(LTC_social) | 7.783 | 1 | 0.0050 |
| Log(GDP pc) | 27.945 | 1 | 0.0000 | Log(GDP pc) | 26.253 | 1 | 0.0000 | Log(GDP pc) | 31.348 | 1 | 0.0000 |
| All | 34.081 | 2 | 0.0000 | All | 26.336 | 2 | 0.0000 | All | 37.826 | 2 | 0.0000 |
| Eq: Log(LTC_total) |  |  |  | Eq: Log(LTC_health) |  |  |  | Eq: Log(LTC_social) |  |  |  |
| Log(Fem_part) | 6.084 | 1 | 0.0140 | Log(Fem_part) | 7.259 | 1 | 0.0070 | Log(Fem_part) | 12.178 | 1 | 0.0000 |
| Log(GDP pc) | 12.796 | 1 | 0.0000 | Log(GDP pc) | 14.904 | 1 | 0.0000 | Log(GDP pc) | 2.895 | 1 | 0.0890 |
| All | 18.021 | 2 | 0.0000 | All | 21.496 | 2 | 0.0000 | All | 15.189 | 2 | 0.0010 |
| Eq: Log(GDP pc) |  |  |  | Eq: Log(GDP pc) |  |  |  | Eq: Log(GDP pc) |  |  |  |
| Log(Fem_part) | 2.89 | 1 | 0.0890 | Log(Fem_part) | 7.921 | 1 | 0.0050 | Log(Fem_part) | 2.256 | 1 | 0.1330 |
| Log(LTC_total) | 8.252 | 1 | 0.0040 | Log(LTC_health) | 0.388 | 1 | 0.5340 | Log(LTC_social) | 8.432 | 1 | 0.0040 |
| All | 16.665 | 2 | 0.0000 | All | 9.111 | 2 | 0.0110 | All | 13.23 | 2 | 0.0010 |
| **Panel-VAR Log(HC_total), Log(LTC_total), Log(GDP pc)** | | | | **Panel-VAR Log(HC_total), Log(LTC_health), Log(GDP pc)** | | | | **Panel-VAR Log(HC_total), Log(LTC_social), Log(GDP pc)** | | | |
| Eq: Log(HC_total) |  |  |  | Eq: Log(HC_health) |  |  |  | Eq: Log(HC_social) |  |  |  |
| Log(LTC_total) | 4.043 | 1 | 0.0440 | Log(LTC_health) | 4.725 | 1 | 0.0300 | Log(LTC_social) | 1.225 | 1 | 0.2680 |
| Log(GDP pc) | 30.129 | 1 | 0.0000 | Log(GDP pc) | 26.885 | 1 | 0.0000 | Log(GDP pc) | 7.868 | 1 | 0.0050 |
| All | 33.239 | 2 | 0.0000 | All | 33.241 | 2 | 0.0000 | All | 10.251 | 2 | 0.0060 |
| Eq: Log(LTC_total) |  |  |  | Eq: Log(LTC_health) |  |  |  | Eq: Log(LTC_social) |  |  |  |
| Log(HC_total) | 0.005 | 1 | 0.9430 | Log(HC_health) | 1.93 | 1 | 0.1650 | Log(HC_social) | 0.536 | 1 | 0.4640 |
| Log(GDP pc) | 2.656 | 1 | 0.1030 | Log(GDP pc) | 0.125 | 1 | 0.7240 | Log(GDP pc) | 1.014 | 1 | 0.3140 |
| All | 2.774 | 2 | 0.2500 | All | 1.953 | 2 | 0.3770 | All | 2.589 | 2 | 0.2740 |
| Eq: Log(GDP pc) |  |  |  | Eq: Log(GDP pc) |  |  |  | Eq: Log(GDP pc) |  |  |  |
| Log(HC_total) | 1.033 | 1 | 0.3100 | Log(HC_health) | 0.003 | 1 | 0.9550 | Log(HC_social) | 5.077 | 1 | 0.0240 |
| Log(LTC_total) | 5.333 | 1 | 0.0210 | Log(LTC_health) | 5.001 | 1 | 0.0250 | Log(LTC_social) | 0.967 | 1 | 0.3250 |
| All | 5.474 | 2 | 0.0650 | All | 5.235 | 2 | 0.0730 | All | 5.243 | 2 | 0.0730 |
| **Panel-VAR Log(Inpatient), Log(LTC_total), Log(GDP pc)** | | | | **Panel-VAR Log(Inpatient), Log(LTC_health), Log(GDP pc)** | | | | **Panel-VAR Log(Inpatient), Log(LTC_social), Log(GDP pc)** | | | |
| Eq: Log(Inpatient) |  |  |  | Eq: Log(Inpatient) |  |  |  | Eq: Log(Inpatient) |  |  |  |
| Log(LTC_total) | 4.817 | 1 | 0.0280 | Log(LTC_health) | 32.047 | 1 | 0.0000 | Log(LTC_social) | 6.507 | 1 | 0.0110 |
| Log(GDP pc) | 16.248 | 1 | 0.0000 | Log(GDP pc) | 33.584 | 1 | 0.0000 | Log(GDP pc) | 20.46 | 1 | 0.0000 |
| All | 16.285 | 2 | 0.0000 | All | 33.903 | 2 | 0.0000 | All | 27.075 | 2 | 0.0000 |
| Eq: Log(LTC_total) |  |  |  | Eq: Log(LTC_health) |  |  |  | Eq: Log(LTC_social) |  |  |  |
| Log(Inpatient) | 0.241 | 1 | 0.6240 | Log(Inpatient) | 1.765 | 1 | 0.1840 | Log(Inpatient) | 34.552 | 1 | 0.0000 |
| Log(GDP pc) | 0.094 | 1 | 0.7590 | Log(GDP pc) | 25.139 | 1 | 0.0000 | Log(GDP pc) | 1.74 | 1 | 0.1870 |
| All | 0.338 | 2 | 0.8440 | All | 25.74 | 2 | 0.0000 | All | 34.692 | 2 | 0.0000 |
| Eq: Log(GDP pc) |  |  |  | Eq: Log(GDP pc) |  |  |  | Eq: Log(GDP pc) |  |  |  |
| Log(Inpatient) | 0.347 | 1 | 0.5560 | Log(Inpatient) | 0.191 | 1 | 0.6620 | Log(Inpatient) | 6.786 | 1 | 0.0090 |
| Log(LTC_total) | 1.639 | 1 | 0.2010 | Log(LTC_health) | 5.418 | 1 | 0.0200 | Log(LTC_social) | 6.849 | 1 | 0.0090 |
| All | 1.996 | 2 | 0.3690 | All | 5.986 | 2 | 0.0500 | All | 14.766 | 2 | 0.0010 |
| **Panel-VAR Log(Outpatient), Log(LTC_total), Log(GDP pc)** | | | | **Panel-VAR Log(Outpatient). Log(LTC_health). Log(GDP pc)** | | | | **Panel-VAR Log(Outpatient). Log(LTC_social). Log(GDP pc)** | | | |
| Eq: Log(Outpatient) |  |  |  | Eq: Log(Outpatient) |  |  |  | Eq: Log(Outpatient) |  |  |  |
| Log(LTC_total) | 0.001 | 1 | 0.9760 | Log(LTC_health) | 6.668 | 1 | 0.0100 | Log(LTC_social) | 36.237 | 1 | 0.0000 |
| Log(GDP pc) | 0.014 | 1 | 0.9050 | Log(GDP pc) | 8.744 | 1 | 0.0030 | Log(GDP pc) | 15.839 | 1 | 0.0000 |
| All | 0.016 | 2 | 0.9920 | All | 13.669 | 2 | 0.0010 | All | 44.124 | 2 | 0.0000 |
| Eq: Log(LTC_total) |  |  |  | Eq: Log(LTC_health) |  |  |  | Eq: Log(LTC_social) |  |  |  |
| Log(Outpatient) | 20.003 | 1 | 0.0000 | Log(Outpatient) | 20.268 | 1 | 0.0000 | Log(Outpatient) | 34.506 | 1 | 0.0000 |
| Log(GDP pc) | 1.213 | 1 | 0.2710 | Log(GDP pc) | 38.491 | 1 | 0.0000 | Log(GDP pc) | 18.347 | 1 | 0.0000 |
| All | 2.051 | 2 | 0.3590 | All | 76.533 | 2 | 0.0000 | All | 50.335 | 2 | 0.0000 |
| Eq: Log(GDP pc) |  |  |  | Eq: Log(GDP pc) |  |  |  | Eq: Log(GDP pc) |  |  |  |
| Log(Outpatient) | 4.845 | 1 | 0.0280 | Log(Outpatient) | 0.569 | 1 | 0.4510 | Log(Outpatient) | 43.924 | 1 | 0.0000 |
| Log(LTC_total) | 0.713 | 1 | 0.3980 | Log(LTC_health) | 2.19 | 1 | 0.1390 | Log(LTC_social) | 9.419 | 1 | 0.0020 |
| All | 7.616 | 2 | 0.0220 | All | 2.951 | 2 | 0.2290 | All | 43.934 | 2 | 0.0000 |
| **Panel-VAR Log(Medicines), Log(LTC_total), Log(GDP pc)** | | | | **Panel-VAR Log(Medicines), Log(LTC_health), Log(GDP pc)** | | | | **Panel-VAR Log(Medicines), Log(LTC_social), Log(GDP pc)** | | | |
| Eq: Log(Medicines) |  |  |  | Eq: Log(Medicines) |  |  |  | Eq: Log(Medicines) |  |  |  |
| Log(LTC_total) | 4.672 | 1 | 0.0310 | Log(LTC_health) | 38.492 | 1 | 0.0000 | Log(LTC_social) | 9.755 | 1 | 0.0020 |
| Log(GDP pc) | 2.746 | 1 | 0.0970 | Log(GDP pc) | 54.737 | 1 | 0.0000 | Log(GDP pc) | 23.467 | 1 | 0.0000 |
| All | 4.778 | 2 | 0.0920 | All | 89.474 | 2 | 0.0000 | All | 27.872 | 2 | 0.0000 |
| Eq: Log(LTC_total) |  |  |  | Eq: Log(LTC_health) |  |  |  | Eq: Log(LTC_social) |  |  |  |
| Log(Medicines) | 2.946 | 1 | 0.0860 | Log(Medicines) | 1.246 | 1 | 0.2640 | Log(Medicines) | 0.054 | 1 | 0.8170 |
| Log(GDP pc) | 0.018 | 1 | 0.8950 | Log(GDP pc) | 2.696 | 1 | 0.1010 | Log(GDP pc) | 2.377 | 1 | 0.1230 |
| All | 4.67 | 2 | 0.0970 | All | 5.33 | 2 | 0.0700 | All | 2.766 | 2 | 0.2510 |
| Eq: Log(GDP pc) |  |  |  | Eq: Log(GDP pc) |  |  |  | Eq: Log(GDP pc) |  |  |  |
| Log(Medicines) | 2.949 | 1 | 0.0860 | Log(Medicines) | 4.143 | 1 | 0.0420 | Log(Medicines) | 0.882 | 1 | 0.3480 |
| Log(LTC_total) | 0.893 | 1 | 0.3450 | Log(LTC_total) | 24.464 | 1 | 0.0000 | Log(LTC_total) | 10.208 | 1 | 0.0010 |
| All | 4.879 | 2 | 0.0870 | All | 27.072 | 2 | 0.0000 | All | 12.525 | 2 | 0.0020 |

The table shows the results of the Granger causality test based on the baseline GMM panel-VAR specification with one lag and one to four lags in the endogenous instruments.

Ho: the excluded variable does not Granger-cause the equation variable.

H1: the excluded variable Granger-causes the equation variable.

The first model suggests that: (i) female labour participation Granger-causes LTC expenditures (total, health, and social), (ii) total LTC expenditures and social LTC expenditures Granger-cause GDP pc, and (iii) social LTC expenditures Granger-causes female labour participation. Moving on to the second model, we find that total LTC expenditures and health LTC expenditures Granger-cause total HCE, as well as inpatient and medication expenditures. In contrast, only the social dimension of LTC expenditures Granger-cause outpatient expenditures. However, outpatient expenditures Granger-cause total LTC expenditures and, finally, inpatient expenditures Granger-cause social LTC expenditures.

As for the Granger-causality of GDP pc, results suggest that: (i) total LTC and health LTC expenditure Granger-cause GDP pc in the model for total HCE, (ii) LTC social expenditure Granger-causes GDP pc in the models for inpatient and outpatient expenditure and (iii) health and social LTC expenditure Granger-cause GDP pc in the model for medicine expenditure. Finally, the condition of stability (i.e., that the module of all of the eigenvalues is within the unit circle) is also verified. For both models, Table B4 shows that this condition is met.

**Table B4. Stability of panel-VAR models. All countries**

|  | Real | Imaginary | Modulus |  | Real | Imaginary | Modulus |  | Real | Imaginary | Modulus |
| --- | --- | --- | --- | --- | --- | --- | --- | --- | --- | --- | --- |
| **Panel-VAR Log(Fem_part), Log(LTC_total), Log(GDP pc)** | | | | **Panel-VAR Log(Fem_part), Log(LTC_health), Log(GDP pc)** | | | | **Panel-VAR Log(Fem_part), Log(LTC_social), Log(GDP pc)** | | | |
|  | | | |  | | | |  | | | |
| Eigenvalue 1 | 0.8053 | 0.0000 | 0.8053 | Eigenvalue 1 | 0.7626 | 0.0000 | 0.7626 | Eigenvalue 1 | 0.8134 | 0.0000 | 0.8134 |
| Eigenvalue 2 | 0.4294 | 0.0000 | 0.4294 | Eigenvalue 2 | 0.4243 | 0.0000 | 0.4243 | Eigenvalue 2 | 0.4079 | 0.0000 | 0.4079 |
| Eigenvalue 3 | 0.1187 | 0.0000 | 0.1187 | Eigenvalue 3 | -0.0307 | 0.0000 | 0.0307 | Eigenvalue 3 | 0.2772 | 0.0000 | 0.2772 |
| **Panel-VAR Log(HC_total), Log(LTC_total), Log(GDP pc)** | | | | **Panel-VAR Log(HC_total), Log(LTC_total), Log(GDP pc)** | | | | **Panel-VAR Log(HC_total), Log(LTC_total), Log(GDP pc)** | | | |
|  | | | |  | | | |  | | | |
| Eigenvalue 1 | 0.8231 | 0.0000 | 0.8231 | Eigenvalue 1 | 0.9192 | 0.0000 | 0.9192 | Eigenvalue 1 | 0.8048 | 0.0000 | 0.8048 |
| Eigenvalue 2 | 0.6622 | -0.1431 | 0.6775 | Eigenvalue 2 | 0.7729 | 0.0000 | 0.7729 | Eigenvalue 2 | 0.2799 | 0.0000 | 0.2799 |
| Eigenvalue 3 | 0.6622 | 0.1431 | 0.6775 | Eigenvalue 3 | 0.6229 | 0.0000 | 0.6229 | Eigenvalue 3 | 0.1769 | 0.0000 | 0.1769 |
| **Panel-VAR Log(Inpatient), Log(LTC_total), Log(GDP pc)** | | | | **Panel-VAR Log(Inpatient), Log(LTC_total), Log(GDP pc)** | | | | **Panel-VAR Log(Inpatient), Log(LTC_total), Log(GDP pc)** | | | |
|  | | | |  | | | |  | | | |
| Eigenvalue 1 | 0.9239 | 0.0000 | 0.9239 | Eigenvalue 1 | 0.6799 | 0.0000 | 0.6799 | Eigenvalue 1 | 0.5475 | -0.0962 | 0.5559 |
| Eigenvalue 2 | 0.8626 | 0.0000 | 0.8626 | Eigenvalue 2 | 0.4789 | 0.0000 | 0.4789 | Eigenvalue 2 | 0.5475 | 0.0962 | 0.5559 |
| Eigenvalue 3 | 0.5757 | 0.0000 | 0.5757 | Eigenvalue 3 | 0.3525 | 0.0000 | 0.3525 | Eigenvalue 3 | -0.1648 | 0.0000 | 0.1648 |
| **Panel-VAR Log(Outpatient), Log(LTC_total), Log(GDP pc)** | | | | **Panel-VAR Log(Outpatient), Log(LTC_total), Log(GDP pc)** | | | | **Panel-VAR Log(Outpatient), Log(LTC_total), Log(GDP pc)** | | | |
|  | | | |  | | | |  | | | |
| Eigenvalue 1 | 0.9164 | 0.0000 | 0.9164 | Eigenvalue 1 | 0.7795 | -0.0606 | 0.7818 | Eigenvalue 1 | 0.6622 | 0.0000 | 0.6622 |
| Eigenvalue 2 | 0.8775 | 0.0000 | 0.8775 | Eigenvalue 2 | 0.7795 | 0.0606 | 0.7818 | Eigenvalue 2 | 0.4281 | 0.0000 | 0.4281 |
| Eigenvalue 3 | 0.6154 | 0.0000 | 0.6154 | Eigenvalue 3 | 0.5610 | 0.0000 | 0.5610 | Eigenvalue 3 | -0.0850 | 0.0000 | 0.0850 |
| **Panel-VAR Log(Medicines), Log(LTC_total), Log(GDP pc)** | | | | **Panel-VAR Log(Medicines), Log(LTC_total), Log(GDP pc)** | | | | **Panel-VAR Log(Medicines), Log(LTC_total), Log(GDP pc)** | | | |
|  | | | |  | | | |  | | | |
| Eigenvalue 1 | 0.6180 | 0.0000 | 0.6180 | Eigenvalue 1 | 0.8783 | 0.0000 | 0.8783 | Eigenvalue 1 | 0.6019 | 0.1622 | 0.6234 |
| Eigenvalue 2 | 0.4802 | 0.1938 | 0.5178 | Eigenvalue 2 | 0.4869 | 0.2225 | 0.5353 | Eigenvalue 2 | 0.6019 | -0.1622 | 0.6234 |
| Eigenvalue 3 | 0.4802 | -0.1938 | 0.5178 | Eigenvalue 3 | 0.4869 | -0.2225 | 0.5353 | Eigenvalue 3 | 0.2506 | 0.0000 | 0.2506 |

Eigenvalues lie inside the unit circle. Panel-VAR for for Model 1 (FemPart, LTC, GDP pc) and Model 2 (LTC, HC, GDP pc) satisfy the stability condition. For both models a GMM panel-VAR with one lag and with one to four lags in the endogenous instruments has been estimated.

|  |  |  |  |
| --- | --- | --- | --- |

**Table B5. Forecast error variance decomposition for Model 1**

| **Forecast horizon** | All countries | | Northern European countries | | Southern European countries | |
| --- | --- | --- | --- | --- | --- | --- |
|  | % | Std.dev | % | Std.dev | % | Std.dev |
| **Impulse: Logfempart**  **Response: LogLTC_total** |  |  |  |  | No signif. |  |
| 0 | 0.0000 | 0.0000 | 0.0000 | 0.0000 | 0.0000 | 0.0000 |
| 1 | 5.7030 | 2.9480 | 13.4440 | 12.8500 | 0.0100 | 7.6040 |
| 2 | 6.4960 | 3.0970 | 22.0340 | 8.1900 | 1.9840 | 6.1950 |
| 3 | 6.9810 | 3.1910 | 36.3160 | 9.0010 | 3.0450 | 6.5830 |
| 4 | 7.3150 | 3.2570 | 50.8320 | 10.1900 | 3.4130 | 6.7280 |
| 5 | 7.5420 | 3.3110 | 62.8380 | 10.9500 | 3.4920 | 6.8390 |
| 6 | 7.6920 | 3.3570 | 70.9070 | 11.0730 | 3.5620 | 7.0090 |
| 7 | 7.7910 | 3.3960 | 75.9080 | 10.8970 | 3.6550 | 7.2150 |
| 8 | 7.8550 | 3.4300 | 78.8270 | 10.6820 | 3.7420 | 7.4170 |
| 9 | 7.8960 | 3.4580 | 80.4530 | 10.4970 | 3.8060 | 7.6100 |
| 10 | 7.9230 | 3.4820 | 81.3370 | 10.3560 | 3.8510 | 7.7880 |
| **Impulse: LogLTC_total**  **Response: LogGDP pc** |  |  |  |  |  |  |
| 0 | 0.0000 | 0.0000 | 0.0000 | 0.0000 | 0.0000 | 0.0000 |
| 1 | 0.1310 | 0.7540 | 1.6540 | 6.3550 | 33.6730 | 15.7340 |
| 2 | 1.8170 | 1.5260 | 8.6330 | 7.1460 | 37.3020 | 14.8370 |
| 3 | 2.5470 | 2.0460 | 8.1390 | 6.5930 | 34.4410 | 13.8460 |
| 4 | 2.8360 | 2.2590 | 8.0280 | 6.0830 | 34.3950 | 13.9660 |
| 5 | 2.9660 | 2.3600 | 8.4300 | 5.5540 | 34.4260 | 13.7590 |
| 6 | 3.0320 | 2.4130 | 8.7720 | 5.1250 | 34.3480 | 13.7760 |
| 7 | 3.0690 | 2.4450 | 9.1180 | 4.9890 | 34.3440 | 13.7260 |
| 8 | 3.0910 | 2.4640 | 9.4910 | 5.1370 | 34.3460 | 13.7000 |
| 9 | 3.1050 | 2.4770 | 9.7970 | 5.4540 | 34.3430 | 13.6830 |
| 10 | 3.1130 | 2.4860 | 10.0080 | 5.8140 | 34.3410 | 13.6770 |
| **Impulse: Logfempart**  **Response: LogLTC_health** |  |  |  |  | No signif. |  |
| 0 | 0.0000 | 0.0000 | 0.0000 | 0.0000 | 0.0000 | 0.0000 |
| 1 | 3.6220 | 0.2120 | 24.6640 | 11.8960 | 14.6260 | 13.5950 |
| 2 | 4.4270 | 0.2930 | 21.0590 | 9.8080 | 10.0970 | 9.6850 |
| 3 | 5.0610 | 0.3560 | 23.7160 | 9.0220 | 9.1980 | 10.0430 |
| 4 | 5.5370 | 0.4040 | 27.7420 | 8.5910 | 9.6080 | 10.4960 |
| 5 | 5.8800 | 0.4380 | 32.1890 | 9.0430 | 10.4920 | 10.8970 |
| 6 | 6.1180 | 0.4620 | 36.8740 | 10.3600 | 11.5500 | 11.2740 |
| 7 | 6.2800 | 0.4780 | 41.7170 | 12.1200 | 12.6500 | 11.6370 |
| 8 | 6.3880 | 0.4890 | 46.6110 | 13.8820 | 13.7290 | 11.9970 |
| 9 | 6.4600 | 0.4960 | 51.4390 | 15.4090 | 14.7590 | 12.3540 |
| 10 | 6.5080 | 0.5010 | 56.0890 | 16.6380 | 15.7230 | 12.7070 |
| **Impulse: LogLTC_social**  **Response: Logfempart** |  |  |  |  |  |  |
| 0 | 0.0000 | 0.0000 | 0.0000 | 0.0000 | 0.0000 | 0.0000 |
| 1 | 0.0000 | 0.0000 | 0.0000 | 0.0000 | 0.0000 | 0.0000 |
| 2 | 0.3840 | 0.0570 | 9.6630 | 11.2810 | 0.3710 | 4.3600 |
| 3 | 0.5250 | 0.0800 | 9.8360 | 16.8900 | 0.4110 | 4.9590 |
| 4 | 0.5940 | 0.0930 | 9.9630 | 20.7470 | 0.4400 | 5.3670 |
| 5 | 0.6300 | 0.1000 | 10.0120 | 23.0690 | 0.4550 | 5.6060 |
| 6 | 0.6490 | 0.1040 | 10.7430 | 24.1420 | 0.4650 | 5.7940 |
| 7 | 0.6600 | 0.1060 | 11.2630 | 24.3120 | 0.4730 | 5.9420 |
| 8 | 0.6660 | 0.1070 | 11.0770 | 24.0680 | 0.4780 | 6.0660 |
| 9 | 0.6690 | 0.1080 | 11.8680 | 23.7230 | 0.4820 | 6.1700 |
| 10 | 0.6710 | 0.1080 | 12.0940 | 23.9000 | 0.4850 | 6.2590 |
| **Impulse: Logfempart**  **Response: LogLTC_social** |  |  |  |  |  |  |
| 0 | 0.0000 | 0.0000 | 0.0000 | 0.0000 | 0.0000 | 0.0000 |
| 1 | 0.9680 | 0.7430 | 1.3860 | 6.5430 | 5.0980 | 7.8140 |
| 2 | 11.5150 | 0.9940 | 15.2710 | 8.6100 | 6.5790 | 8.7030 |
| 3 | 14.4700 | 1.1240 | 30.8310 | 14.1310 | 6.4240 | 8.3780 |
| 4 | 15.6080 | 1.2050 | 43.8290 | 18.3320 | 7.0100 | 8.4700 |
| 5 | 16.1070 | 1.2560 | 54.4010 | 20.8370 | 7.9540 | 8.6710 |
| 6 | 16.3500 | 1.2900 | 63.0010 | 22.4050 | 9.1300 | 8.9990 |
| 7 | 16.4760 | 1.3140 | 70.0030 | 24.1800 | 10.4520 | 9.4340 |
| 8 | 16.5460 | 1.3310 | 75.7110 | 25.4360 | 11.8720 | 9.9760 |
| 9 | 16.5850 | 1.3450 | 80.3670 | 26.7840 | 13.3570 | 10.6030 |
| 10 | 16.6070 | 1.3550 | 84.1690 | 27.5190 | 14.8870 | 11.2960 |
| **Impulse: LogLTC_social**  **Response: LogGDP pc** |  |  |  |  |  |  |
| 0 | 0.0000 | 0.0000 | 0.0000 | 0.0000 | 0.0000 | 0.0000 |
| 1 | 4.3380 | 2.5610 | 11.5920 | 2.0530 | 3.0180 | 1.5100 |
| 2 | 4.3400 | 1.9260 | 11.7910 | 1.9840 | 3.0780 | 2.4820 |
| 3 | 4.3430 | 1.8420 | 11.9990 | 2.0100 | 3.0560 | 2.4810 |
| 4 | 4.3490 | 1.8130 | 12.2120 | 2.2950 | 3.0510 | 2.4790 |
| 5 | 4.3590 | 1.7990 | 12.4270 | 2.8250 | 3.0470 | 2.4780 |
| 6 | 4.3770 | 1.7910 | 12.6380 | 3.3440 | 3.0450 | 2.4780 |
| 7 | 4.4140 | 1.7870 | 12.8400 | 3.8170 | 3.0430 | 2.4790 |
| 8 | 4.4980 | 1.7850 | 13.0350 | 4.1180 | 3.0420 | 2.4790 |
| 9 | 4.7420 | 1.7840 | 13.1020 | 4.3160 | 3.0410 | 2.4800 |
| 10 | 5.7300 | 1.7830 | 13.4390 | 4.4500 | 3.0400 | 2.4810 |

The table shows the variation in the response variable explained by the impulse variable. Model 1 (FemPart, LTC, GDP pc). Estimation of GMM panel-VAR for both models with one lag and one to four lags in the endogenous instruments has been made. The variance decomposition is at a horizon of 10 years after the shock. The ordering of the variables in the Cholesky decomposition for Model 1 is as follows: Female labour participation 🡪 LTC expenditures🡪 GDP pc.

**Table B6. Forecast error variance decomposition for Model 2**

| **Forecast horizon** | All countries | | Northern European countries | | Southern European countries | |
| --- | --- | --- | --- | --- | --- | --- |
|  | % | Std.dev | % | Std.dev | % | Std.dev |
| **Impulse: LogHC_total**  **Response: LogLTC_total** | No signif. |  |  |  |  |  |
| 0 | 0.0000 | 0.0000 | 0.0000 | 0.0000 | 0.0000 | 0.0000 |
| 1 | 0.2548 | 1.1827 | 0.0000 | 0.0000 | 0.8042 | 0.4012 |
| 2 | 2.0144 | 1.6014 | 0.2744 | 0.1938 | 0.5923 | 0.3348 |
| 3 | 3.3066 | 1.6910 | 0.7907 | 0.3974 | 1.0246 | 0.4555 |
| 4 | 3.9097 | 1.7167 | 1.4499 | 0.5326 | 1.6017 | 0.5541 |
| 5 | 4.1037 | 1.7237 | 2.1833 | 0.6173 | 2.1376 | 0.6132 |
| 6 | 4.1123 | 1.7240 | 2.9438 | 0.6718 | 2.5768 | 0.6484 |
| 7 | 4.0566 | 1.7220 | 3.6996 | 0.7085 | 2.9149 | 0.6701 |
| 8 | 3.9927 | 1.7197 | 4.4297 | 0.7342 | 3.1653 | 0.6839 |
| 9 | 3.9421 | 1.7179 | 5.1210 | 0.7530 | 3.3460 | 0.6929 |
| 10 | 3.9092 | 1.7167 | 5.7657 | 0.7670 | 3.4738 | 0.6988 |
| **Impulse: LogLTC_total**  **Response: LogHC_total** |  |  |  |  |  |  |
| 0 | 0.0000 | 0.0000 | 0.0000 | 0.0000 | 0.0000 | 0.0000 |
| 1 | 0.0000 | 0.0000 | 0.4073 | 0.2605 | 0.0000 | 0.0000 |
| 2 | 1.1736 | 0.4859 | 1.5599 | 0.5484 | 0.7298 | 0.3797 |
| 3 | 1.3873 | 0.6949 | 3.2640 | 0.6889 | 1.6327 | 0.5581 |
| 4 | 1.9834 | 0.7711 | 5.3813 | 1.7590 | 2.4327 | 0.6378 |
| 5 | 2.4540 | 0.8048 | 7.7672 | 1.7973 | 3.0706 | 0.6789 |
| 6 | 2.5512 | 0.8221 | 10.2854 | 2.8203 | 3.5544 | 1.0024 |
| 7 | 2.2171 | 0.8319 | 12.8183 | 2.8349 | 3.9108 | 1.1167 |
| 8 | 3.4875 | 1.5379 | 15.2733 | 2.8447 | 4.1685 | 1.7259 |
| 9 | 4.4310 | 1.7417 | 17.5841 | 2.8516 | 4.3524 | 1.7319 |
| 10 | 5.1188 | 1.8442 | 19.7086 | 2.8565 | 4.4828 | 1.7358 |
| **Impulse: LogLTC_total**  **Response: LogGDP pc** |  |  |  |  |  |  |
| 0 | 0.0000 | 0.0000 | 0.0000 | 0.0000 | 0.0000 | 0.0000 |
| 1 | 2.9785 | 0.6738 | 0.2552 | 0.1830 | 0.1461 | 0.1147 |
| 2 | 4.9339 | 0.7483 | 1.8535 | 0.5846 | 1.1005 | 0.0822 |
| 3 | 7.1417 | 0.7895 | 4.5245 | 0.7371 | 2.0855 | 0.0709 |
| 4 | 9.4091 | 0.8135 | 7.9434 | 0.7994 | 3.0871 | 0.0721 |
| 5 | 11.5818 | 0.8285 | 11.7255 | 0.8293 | 5.1049 | 1.0855 |
| 6 | 13.5566 | 0.8382 | 15.5319 | 2.8456 | 7.1383 | 1.1093 |
| 7 | 15.2793 | 0.8447 | 19.1234 | 3.8553 | 8.1842 | 2.1400 |
| 8 | 16.7331 | 0.8492 | 22.3647 | 3.8615 | 10.2379 | 3.1730 |
| 9 | 17.9275 | 0.8525 | 25.2018 | 4.8657 | 13.2946 | 3.2048 |
| 10 | 18.8867 | 0.8547 | 27.6335 | 4.8686 | 15.3501 | 3.2334 |
| **Impulse: LogHC_total**  **Response: LogGDP pc** | No signif. |  |  |  |  |  |
| 0 | 0.0000 | 0.0000 | 0.0000 | 0.0000 | 0.0000 | 0.0000 |
| 1 | 0.6001 | 1.3375 | 3.0292 | 0.6766 | 0.0018 | 0.0016 |
| 2 | 0.9135 | 1.4296 | 2.8156 | 0.6641 | 0.2237 | 0.1645 |
| 3 | 1.2191 | 1.4944 | 2.5515 | 0.6466 | 0.6421 | 0.3519 |
| 4 | 1.4940 | 1.5391 | 2.3045 | 0.6276 | 1.1435 | 0.4801 |
| 5 | 1.7279 | 1.5701 | 2.1272 | 0.6122 | 1.6490 | 0.5603 |
| 6 | 1.9194 | 1.5917 | 2.0475 | 0.6047 | 2.1128 | 0.6109 |
| 7 | 2.0717 | 1.6070 | 2.0705 | 0.6069 | 2.5134 | 0.6438 |
| 8 | 2.1904 | 1.6179 | 2.1856 | 0.6175 | 2.8456 | 0.6660 |
| 9 | 2.2814 | 1.6257 | 2.3746 | 0.6333 | 3.1129 | 0.6812 |
| 10 | 2.3505 | 1.6314 | 2.6172 | 0.6512 | 3.3235 | 0.6918 |
| **Impulse: LogLTC_total**  **Response: LogInpatient** |  |  |  |  |  |  |
| 0 | 0.0000 | 0.0000 | 0.0000 | 0.0000 | 0.0000 | 0,0000 |
| 1 | 0.1053 | 0.0858 | 1.4094 | 0.5265 | 1.6688 | 0,5628 |
| 2 | 1.0663 | 0.4644 | 5.5122 | 0.7618 | 2.4842 | 0,6417 |
| 3 | 2.0328 | 0.6032 | 11.7487 | 1.8294 | 2.8342 | 0,6653 |
| 4 | 2.7534 | 0.6602 | 18.1813 | 2.8531 | 2.8606 | 0,6669 |
| 5 | 3.2344 | 0.6875 | 23.7065 | 2.8636 | 2.7356 | 0,6591 |
| 6 | 3.5388 | 0.7017 | 28.1864 | 3.8692 | 2.5770 | 0,6484 |
| 7 | 3.7237 | 0.7095 | 31.8058 | 3.8726 | 2.4540 | 0,6394 |
| 8 | 3.8307 | 0.7137 | 34.7657 | 4.8748 | 2.4021 | 0,6355 |
| 9 | 3.8882 | 0.7159 | 37.2191 | 5.8765 | 2.4354 | 0,6380 |
| 10 | 3.9150 | 0.7169 | 39.2749 | 5.8777 | 2.5536 | 0,6467 |
| **Impulse: LogLTC_total**  **Response: LogOutpatient** | No signif. |  |  |  |  |  |
| 0 | 0.0000 | 0.0000 | 0.0000 | 0.0000 | 0.0000 | 0,0000 |
| 1 | 1.2583 | 1.5015 | 0.0539 | 0.0461 | 8.7592 | 0,8078 |
| 2 | 1.2326 | 1.4969 | 0.3027 | 0.2091 | 6.4988 | 0,7800 |
| 3 | 1.2139 | 1.4935 | 0.7565 | 0.3876 | 4.8160 | 0,7453 |
| 4 | 1.2003 | 1.4910 | 1.4286 | 0.5294 | 3.5640 | 0,7028 |
| 5 | 1.1903 | 1.4891 | 2.3204 | 0.6289 | 2.6360 | 0,6525 |
| 6 | 1.1830 | 1.4877 | 3.4240 | 0.6966 | 1.9524 | 0,5952 |
| 7 | 1.1776 | 1.4867 | 4.7243 | 1.7428 | 1.4531 | 0,5331 |
| 8 | 1.1736 | 1.4859 | 6.2009 | 1.7750 | 1.0930 | 0,4700 |
| 9 | 1.1706 | 1.4854 | 7.8302 | 2.7981 | 2.8380 | 0,4103 |
| 10 | 1.1684 | 1.4849 | 9.5865 | 3.8150 | 2.6624 | 0,5586 |
| **Impulse: LogLTC_total**  **Response: LogMedicines** |  |  |  |  |  |  |
| 0 | 0.0000 | 0.0000 | 0.0000 | 0.0000 | - | - |
| 1 | 0.5801 | 0.3304 | 0.4923 | 0.2969 | - | - |
| 2 | 2.8228 | 0.6646 | 3.0371 | 0.6771 | - | - |
| 3 | 3.0686 | 0.6788 | 6.2127 | 0.7752 | - | - |
| 4 | 2.6613 | 0.6542 | 9.2299 | 0.8120 | - | - |
| 5 | 2.2530 | 0.6233 | 11.7520 | 2.8294 | - | - |
| 6 | 1.9668 | 0.5966 | 13.7233 | 3.8389 | - | - |
| 7 | 1.7899 | 0.5774 | 15.2083 | 4.8445 | - | - |
| 8 | 1.6873 | 0.5651 | 16.3035 | 5.8480 | - | - |
| 9 | 1.6303 | 0.5578 | 17.1016 | 5.8503 | - | - |
| 10 | 1.5994 | 0.5538 | 17.6790 | 5.8518 | - | - |

The table shows the variation in the response variable explained by the impulse variable. Model 2 (LTC, HC, GDP pc). Estimation of GMM panel-VAR for both models with one lag and one to four lags in the endogenous instruments has been made. The variance decomposition is at a horizon of 10 years after the shock. The ordering of the variables in the Cholesky decomposition for Model 2 is: LTC expenditures 🡪 HC expenditures 🡪 GDP pc.
